# Polymer‐in‐Cage Strategy for Pore Tuning of High‐Aspect Ratio ZIF Nanoplate: Toward Sub‐Micrometer‐Thick Large Area CO_2_ Separation Membranes

**DOI:** 10.1002/advs.202519351

**Published:** 2026-02-16

**Authors:** Minsu Kim, Hyo Jun Min, Min Kyu Choi, Ki Chul Kim, Bomi Kim, Miso Kang, Nahyeon Lee, Kiwon Eum, Jong Hak Kim, Dae Woo Kim

**Affiliations:** ^1^ Department of Chemical and Biomolecular Engineering Yonsei University Seoul Republic of Korea; ^2^ Hydrogen Energy Research Division Korea Institute of Energy Research Daejeon Republic of Korea; ^3^ Department of Chemical Engineering Konkuk University Seoul Republic of Korea; ^4^ Green Carbon Research Center Chemical and Process Technology Division Korea Research Institute of Chemical Technology Daejeon Republic of Korea; ^5^ Department of Chemical Engineering Soongsil University Seoul Republic of Korea

**Keywords:** carbon dioxide capture, comb copolymers, gas separation, high aspect ratio, mixed matrix membranes, pore tuning, thin film composite membranes

## Abstract

Mixed‐matrix membranes (MMMs) offer a promising route for CO_2_ separation, yet their potential is often limited by poor polymer‐filler interfaces and challenges in integrating high‐aspect‐ratio fillers into scalable, defect‐free thin‐film composite (TFC) membranes. Here, we introduce a “polymer‐in‐cage” strategy that addresses these issues in a single casting step. A custom‐synthesized comb‐shaped copolymer (PZO) containing zinc‐ion sites is designed to function dually as a mechanically robust matrix and an active pore‐modulating agent for high‐aspect‐ratio ZIF‐8 nanoplates (NZIF‐8). The copolymer's Zn^2+^‐acrylate sites electrostatically anchor into the ZIF‐8 pore windows, constricting their flexible apertures to enhance molecular sieving. The resulting TFC membranes exhibit an exceptional CO_2_/N_2_ selectivity of 80 and a CO_2_ permeance of 333 GPU. This performance stems from a dual enhancement, where polymer‐induced pore tuning is amplified by the tortuous diffusion pathways created by the aligned nanoplates. Furthermore, the membranes demonstrate excellent operational durability under high‐pressure, humid, and long‐term conditions. By uniquely integrating polymer chemistry with MOF architecture, this scalable strategy offers a new design paradigm for fabricating next‐generation membranes for CO_2_ capture and other critical separations.

## Graphical abstract

A “polymer‐in‐cage” strategy transforms a zinc‐acrylate copolymer into an active pore‐modulating agent for ZIF‐8 nanoplates. The polymer actively tunes the MOF's pore structure by anchoring within its windows, while the nanoplates create tortuous pathways. This single‐step fabrication yields defect‐free thin‐film composite membranes with exceptional CO_2_/N_2_ selectivity, offering a scalable route to advanced gas separation.

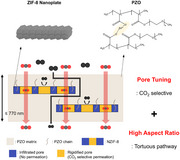

## 1 Introduction

Membrane separation has gained significant attention as an energy‐efficient technology for CO_2_ capture in post‐combustion flue gas treatment and natural gas sweetening, offering lower energy requirements, smaller footprints, and easier modular scale‐up compared to conventional absorption or cryogenic processes [1]. However, preparing membranes with both high CO_2_ permeability and selectivity in a robust, scalable membrane structure remains challenging, especially when targeting industrially relevant separation performance at practical pressures and compositions [2]. Among the various types of membranes, mixed‐matrix membranes (MMMs), which combine the processability of polymers with the molecular sieving capability of porous fillers, have emerged as a promising approach to overcome the performance limitations of polymer membranes [3]. In particular, metal–organic frameworks (MOFs), with their tunable microporosity and thermal/chemical stability, have been widely explored as filler materials [4]. Nonetheless, conventional MOF particles are typically incorporated at relatively low loadings, particularly in thin‐film composite (TFC) structures, which restricts their contribution to enhancing selective transport [5, 6]. In addition, their isotropic morphology limits efficient packing within the thin selective layers essential for practical membranes.

Recently, high‐aspect‐ratio porous nanosheets have been utilized for membrane fabrication because they can provide extended diffusion pathways and enhanced alignment within a polymer matrix, thereby improving molecular selectivity [7, 8, 9, 10, 11, 12, 13, 14]. In particular, MOFs with high aspect ratios have been highlighted as effective materials for improving the performance of gas separation membranes. High‐aspect‐ratio MOFs can provide tortuous pathways for non‐permeable molecules, while permeable gases easily permeate through. Therefore, overall gas selectivity can be significantly enhanced. High‐aspect‐ratio materials can also improve the mechanical strength of the resulting MMM, enabling the fabrication of high‐loading membranes. The layered structure of high‐aspect‐ratio materials can reduce the formation of defects and imperfections in the membrane compared to when isotropic particles are incorporated, especially when the thickness of the selective layer is decreased to the sub‐micrometer scale.

Despite the merits of high‐aspect‐ratio MOF nanosheets in enhancing gas selectivity, their low yield, complex synthesis, and limited types of crystalline structure hinder their integration into scalable membranes for critical applications like CO_2_ capture. For instance, the pioneering work by Rodenas et al. first demonstrated that incorporating bottom‐up synthesized CuBDC nanosheets into a polymer matrix created tortuous diffusion pathways, leading to a significant enhancement in CO_2_/CH_4_ selectivity [15]. Subsequent studies have explored various strategies, such as combining 2D nanosheets with 3D MOF particles to simultaneously tailor both permeability and selectivity, or designing NTU‐82 nanosheets to promote faster CO_2_ transport [16, 17]. Here, we report a polymer‐in‐cage strategy using a zinc‐containing comb‐shaped copolymer (PZO) to serve as both polymer matrix and pore‐tuning agent of ZIF‐8 nanoplates (NZIF‐8) in a single casting step. ZIF‐8 was selected for its well‐defined pore architecture, thermal stability, and gate‐opening behavior, but its 3.4–4.0 Å aperture limits CO_2_/N_2_ selectivity by offering poor size discrimination between CO_2_ (3.3 Å) and N_2_ (3.64 Å). By confining PZO within the NZIF‐8 cages, we rigidified the framework and narrowed the effective pore size, overcoming the intrinsic flexibility of ZIF‐8. Notably, instead of using isotropic ZIF‐8 particles, NZIF‐8s were selected as the filler material. This choice directly addresses the scalability challenge of high‐aspect‐ratio fillers, as these nanoplates can be produced in bulk powder via a practical conversion method. This approach enables the formation of defect‐free, submicron‐thick TFC membranes over large areas, with aligned NZIF‐8 providing both high‐aspect‐ratio‐induced diffusion selectivity and polymer‐driven pore tuning. As a result, the resulting membranes exhibit exceptional CO_2_/N_2_ selectivity and high permeance through a scalable and simple fabrication method. This work offers a new path toward designing functional fillers and next‐generation gas separation membranes for practical thin film membrane applications.

## 2 Results and Discussion

### 2.1 Design and Implementation of the Polymer‐in‐Cage Strategy

#### 2.1.1 Polymer Design for the ZIF‐8 Nanoplate (NZIF‐8) Pore Tuning

**FIGURE 1:**
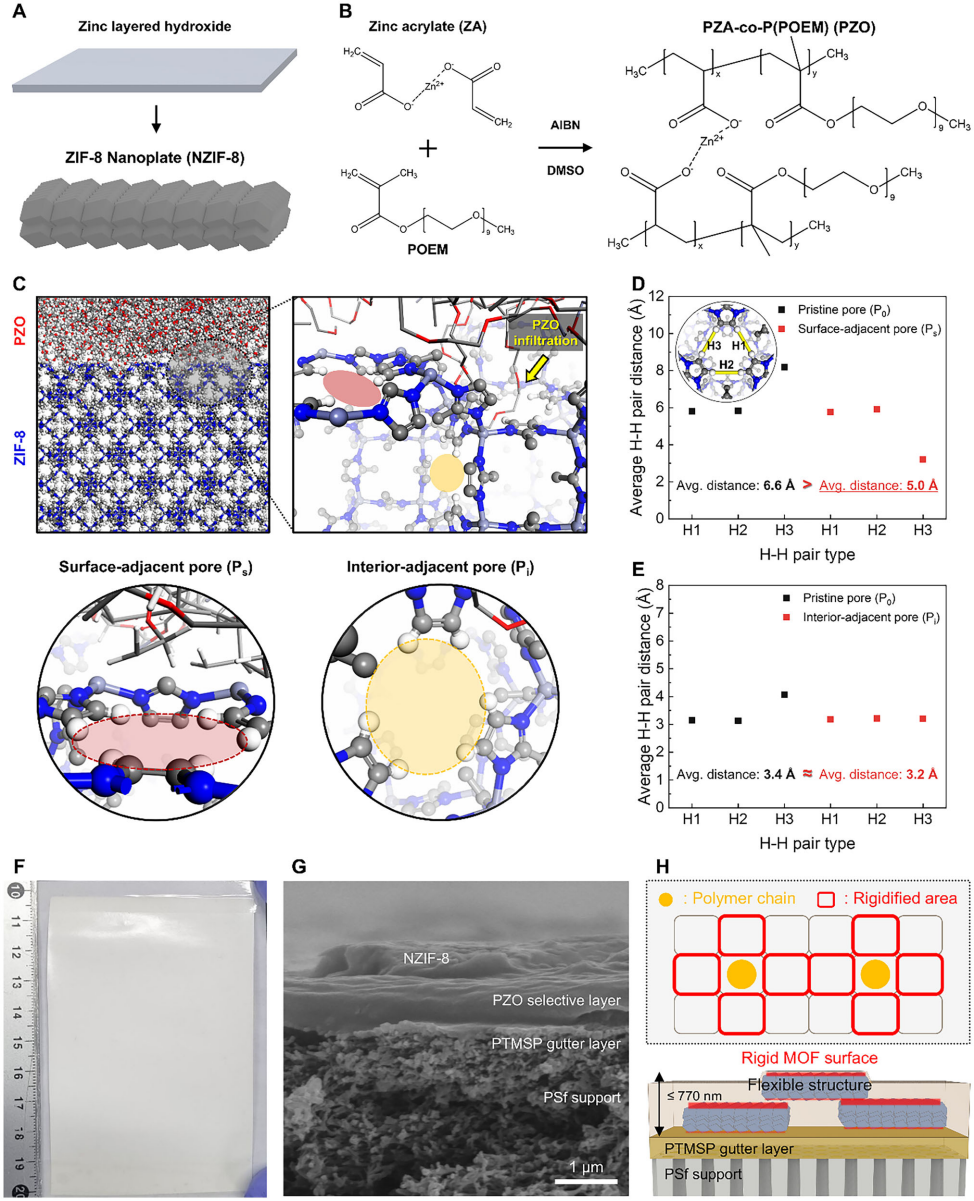
Polymer‐in‐cage strategy in PZZ TFC MMM. Synthesis procedure of NZIF‐8 (A) and structure of PZO (B). (C) Molecular simulation of PZO/ZIF‐8 interface along with snapshots of surface‐adjacent pore (P_s_) and interior‐adjacent pore (P_i_), which is neighboring with PZO entangled or infiltrated pore. (D, E) The average H–H pair distance of P_s_ (D) and P_i_ (E) before and after PZO entanglement & infiltration, indicating a constriction effect induced by PZO. The three distinct H‐H pairs (H1, H2, and H3) within the six‐membered ring define the local pore size. (F) Photographic image of PZZ‐10 TFC MMM. (G) Cross‐sectional SEM image of PZZ‐10 TFC MMM. H, Schematic illustration of the cross‐section structure of PZZ TFC MMM and simplified PZO infiltrated ZIF‐8 model, representing restricted linker mobility in P_s_.

NZIF‐8 was synthesized by the template‐assisted conversion method as previously reported (Figure 1A; Figure S1) [11]. First, a high‐aspect‐ratio Zn_5_(NO_3_)_2_OH_8_ nanosheet was synthesized as a precursor material through an ambient temperature aqueous process (Figure S2A). The intrinsic morphology of the precursor allows for the retention of a high aspect ratio even after the conversion process. However, controlling crystal growth is critical, as it can induce increased thickness or break the nanosheet, thereby decreasing the aspect ratio. Therefore, after carefully selecting the precursor, synthesis conditions such as solvent, concentration, and reaction time were optimized. Specifically, an acetone‐mediated conversion was employed, which allowed for minimal damage to the nanosheet morphology, successfully yielding NZIF‐8 with a high aspect ratio of approximately 20 (Figure S2B). For a comparative study, isotropic ZIF‐8 (IZIF‐8) with an aspect ratio of 1 was also synthesized to a similar thickness of ∼200 nm to specifically investigate the lateral effect (Figure S3). Both NZIF‐8 and IZIF‐8 exhibited identical crystal structures with SOD topology, matching the simulated pattern (Figure S4). Furthermore, N_2_ adsorption‐desorption analysis confirmed their identical microporous structures, showing type I isotherms and similar pore size distributions (Figure S5). The large meso/macropore volume observed in IZIF‐8 is likely due to interparticle spacing, whereas its absence in NZIF‐8 suggests a tight stacking of crystallites with minimal grain‐boundary voids.

Previous methods to tune the pore structure of ZIFs, such as ligand exchange or post‐synthetic treatments, are often difficult to apply to fragile polycrystalline nanoplate morphologies [18, 19, 20, 21, 22]. To overcome these limitations, we developed an alternative strategy by inserting a polymeric pore‐modulating agent to the MOF apertures. For this purpose, a metal‐ion‐containing comb‐shaped copolymer, poly(zinc acrylate)‐co‐poly(oxyethylene methacrylate) (PZA‐co‐POEM), PZO), was synthesized via a low‐cost, commercially available free‐radical polymerization process (Figure 1B). This copolymer was designed to function both as a CO_2_‐selective polymer matrix and as a pore‐modulating agent. Zinc acrylate (ZA) and poly(oxyethylene methacrylate) (POEM) were selected as monomers. POEM, an amorphous poly(ethylene oxide), exhibits excellent CO_2_ separation properties owing to strong quadrupole–dipole interactions between CO_2_ and its ether groups. However, it suffers from liquid‐like mechanical weakness [23]. In contrast, the Zn^2+^ ions in ZA function as ionic crosslinkers, reinforcing the mechanical integrity of the copolymer and overcoming the shortcomings of POEM‐based polymers [24]. Consequently, whereas the homopolymer derived solely from POEM macromonomers remains liquid‐like, the PZO copolymer forms a mechanically robust solid. Moreover, Zn^2+^ ions can form π‐complexes with CO_2_, acting as carriers to facilitate CO_2_ transport even under dry conditions [25, 26]. These combined attributes render the PZO comb‐shaped copolymer a highly promising and multifunctional polymeric matrix for CO_2_ separation applications.

The successful synthesis of the PZO comb‐shaped copolymer was validated by multiple characterization techniques. In FT‐IR analysis, the complete disappearance of the C═C double bond stretching vibrations, originally observed around 1638 cm^−1^ (POEM) and 1649 cm^−1^ (ZA), confirmed the successful polymerization. Moreover, the characteristic sharp bands of the zinc carboxylate group in the ZA monomer (1594 and 1439 cm^−1^) broadened and shifted to higher wavenumbers (1599 and 1454 cm^−1^) in the PZO copolymer, indicating steric hindrance from the polymer chains (Figure S6) [27, 28]. ^1^H‐NMR analysis confirmed that the PZA content in the PZO copolymer was 33.9% (Figure S7), closely matching the initial monomer feed ratio of 37.5%, thereby demonstrating the reliable tunability of copolymer composition via free radical polymerization. Notably, after solvent removal, the PZO copolymer becomes insoluble in common solvents, attributable to strong ionic crosslinking between PZA chains, thereby confirming the formation of a physically crosslinked network. Finally, EDS analysis was conducted to confirm the presence and quantity of Zn ions. As shown in Figure S8 and Table S1, Zn was clearly present in the PZO copolymer with an atomic ratio of 2.19%, which is in reasonable agreement with the value calculated from ^1^H‐NMR analysis (1.29%). Taken together, these results confirm that the zinc‐ion‐containing PZO copolymer was successfully synthesized as designed. The ionic groups in PZO copolymer are expected to create strong electrostatic interactions with ZIF‐8, enabling both precise pore tuning and excellent interfacial compatibility, which are critical for fabricating high‐performance TFC mixed‐matrix membranes.

#### 2.1.2 Molecular Dynamics (MD) Simulations to Reveal PZO‐Induced Pore Tuning of NZIF‐8

MD simulations of the ZIF‐8/PZO interface reveal strong electrostatic interactions between the Zn^2+^‐acrylate sites of PZO and the 2‐methylimidazolate linkers of ZIF‐8. The six‐membered ring architecture formed by 2‐methylimidazolate linkers in ZIF‐8 creates a pore size of 3.4 Å [29]. This small pore size often plays a crucial role in controlling the connectivity between Zn(II) centers and determining the molecular sieving performance of ZIF‐8. MD simulations were employed to identify pore sizes at three distinct pore sites, which could be determined by averaging distances of three H‐H pairs formed by six 2‐methylimidazolate linkers in a six‐membered ring of ZIF‐8. Visual inspection of equilibrated trajectories reveals that, beyond simply adhering to the surface, some PZO chain termini infiltrate into ZIF‐8 pores (Figure 1C). These infiltrated pores become partially blocked by the polymer, resulting in measurable decreases in the average H‐H distances. We define three pore categories; the distinct pore sites are classified as pristine pore (P_0_), surface‐adjacent pore (P_s_), and interior‐adjacent pore (P_i_). P_0_ is a typical pore of ZIF‐8, without any interaction or presence with PZO. P_s_ denotes a surface pore directly adjacent to an infiltrated site, and P_i_ denotes an interior pore one layer below the surface, indirectly affected by infiltration.

Notably, three distinct H‐H pairs in each pore site are labeled by H1, H2, and H3. The pristine ZIF‐8 surface model is simulated to have pore sizes around 3.4 to 6.0 Å while having a uniform pore size of 3.4 Å in the bulk phase, which is consistent with experimentally reported pore sizes, validating the accuracy of our simulation approach in the structural characterization of ZIF‐8 surface models. However, the infiltration of PZO copolymer chains into the ZIF‐8 surfaces modulates the distances of the three H‐H pairs in the infiltrated pore, exhibiting a decrease in the pore sizes from 3.4 to 3.0 Å, resulting from the slight constriction of organic ligands in the penetration pore (Figure S9). The resulting pore reduction likely affects not only the infiltrated pore but also the nearest neighboring pores, potentially modifying overall pore architecture. Specifically, the H‐H distance of P_s_ is reduced significantly from 6.6 Å (pristine ZIF‐8 surface) to 5.0 Å, in contrast to a slight decrease in H‐H distance for P_i_ from 3.4 to 3.2 Å, suggesting a less pronounced structural impact of neighboring subsurface site relative to neighboring surface site (Figure 1D,E). These findings demonstrate that PZO copolymer infiltration induces pore rigidification in ZIF‐8 (sub)surfaces by restricting linker mobility, which may positively influence its molecular sieving effects through the modulation of the selectivity in CO_2_ separation applications.

#### 2.1.3 Fabrication of Sub‐Micron TFC Membranes

The NZIF‐8/PZO (PZZ) mixed‐matrix membranes were fabricated via a simple bar‐coating process from a single casting solution of the PZO copolymer and NZIF‐8 (Figure S10). This selective layer was formed on a porous polysulfone (PSf) support equipped with a PTMSP gutter layer. Since both PSf and PTMSP are insoluble in the ethanol‐water medium, their porous structures remained intact during coating [30, 31, 32]. Large‐area TFC MMMs (over 6 cm x 10 cm) were successfully prepared (Figure 1F). Shear forces during bar‐coating induced horizontal alignment of the NZIF‐8s, even within the submicron‐thick selective layer (Figure 1G) [11, 33]. The average thickness of the TFC‐MMMs ranged from approximately 370 to 770 nm, increasing with NZIF‐8 loading due to the volume occupied by the filler. Importantly, the membranes remained defect‐free, owing to strong interfacial interactions between the PZO copolymer and NZIF‐8 that promote uniform dispersion and tight polymer coverage. Additionally, the inherent adhesive properties of the POEM segments are known to promote excellent film‐forming characteristics, likely contributing to the uniform and intimate coating of the PZO solution onto the support [23, 34, 35]. Together, these factors enable the formation of robust, continuous composite layers even when the local thickness is larger than the polymer selective layer (Figure S11).

As previously described, the PZO comb‐shaped copolymer exhibits robust mechanical properties due to the ionic crosslinking of PZA chains. Unlike previously reported POEM‐based copolymers with a jelly‐like consistency, the PZO copolymer maintains excellent mechanical properties even with a high ratio of POEM chains. Figure S12 shows photographic images of robust and flexible free‐standing dense PZZ membranes with different NZIF‐8 loadings. With increasing NZIF‐8 content, the membranes became less transparent due to refractive index differences between the two phases. The mechanical properties of the PZZ membranes were investigated using universal tensile testing (Figure S13). The neat PZO copolymer exhibited an elongation at break of 150% at a tensile strength of 3.5 MPa. While the incorporation of particles typically reduces membrane flexibility, the tensile stress and strain of PZZ‐5 and PZZ‐10 membranes remained almost the same as those of the neat PZO copolymer. This is likely because NZIF‐8 particles interrupt some of the PZA physical crosslinking while simultaneously maintaining strong interactions with the polymer matrix. At higher NZIF‐8 loadings (15 and 20 wt.%), the tensile strength increased to over 5 MPa while elongation decreased, a behavior typically observed for particle‐filled MMMs. These results demonstrate that the PZZ membranes possess sufficient mechanical integrity for practical gas separation applications.

Finally, as illustrated in the schematic (Figure 1H), the resulting TFC‐MMM adopts a stratified architecture in which NZIF‐8s are normally stacked one to two layers within the submicron selective layer on the PTMSP gutter and PSf support. Given the 200–300 nm thickness of individual NZIF‐8s, a maximum stacking of three layers and predominantly one to two layers is expected, consistent with the observed film thickness remaining below 770 nm even at the highest NZIF‐8 loading. In the schematic, the red‐outlined pores above and below each NZIF‐8 denote the regions where PZO chains have infiltrated and rigidified the pore windows. Upon infiltration, the polymer chains occupy and anchor to the six‐membered ring apertures, restricting linker mobility in adjacent pores (highlighted in red) and contracting the effective aperture toward the intrinsic 3.4 Å size rather than the swing‐expanded 4.0 Å (Figure S14). This polymer‐induced rigidification of surface pores underpins the enhanced molecular sieving behavior and the high CO_2_/N_2_ selectivity observed in subsequent gas separation experiments.

### 2.2 Experimental Confirmation of Polymer‐Induced Pore Tuning in PZZ TFC MMM

#### 2.2.1 Polymer‐Dependent Selective Pore Tuning in TFC MMMs

**FIGURE 2:**
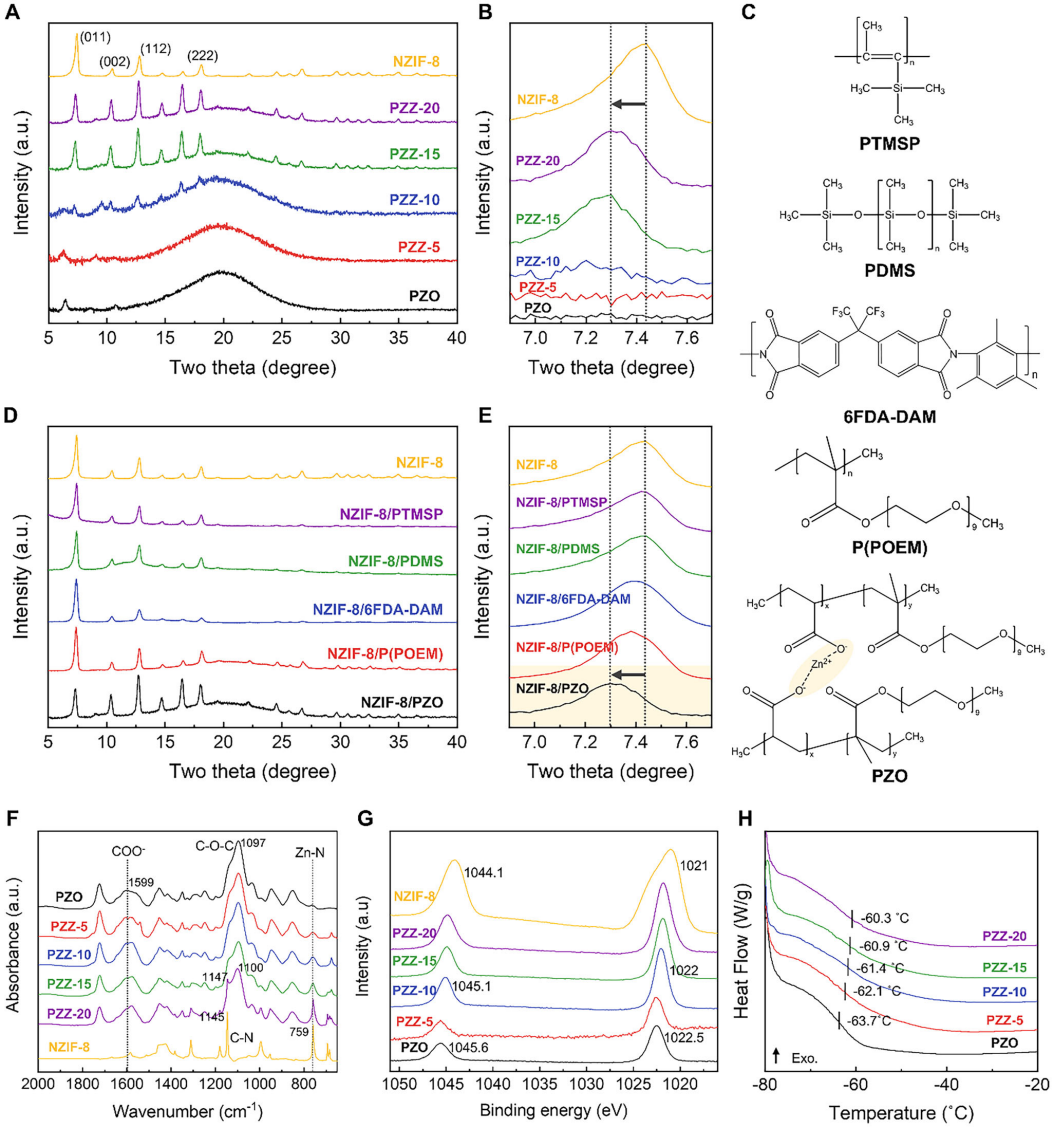
Characterization of polymer‐induced pore tuning in PZZ TFC MMM. (A) XRD patterns of PZO copolymer, NZIF‐8, and PZZ membranes with different loadings of NZIF‐8. (B), Magnified XRD patterns of (011) peak. (C) Chemical structures of the polymers (PTMSP, PDMS, 6FDA‐DAM, P(POEM), and PZO) used for the fabrication of MMMs. (D) XRD patterns of NZIF‐8‐based MMM with various polymers. (E) Magnified XRD patterns of the (011) peak. Pore‐tuning polymer is marked with a yellow box. FT‐IR spectra (F), XPS analysis in Zn 2p region (G), DSC curves (H) of PZO copolymer and PZZ membranes.

XRD analysis was performed to examine the crystalline structure of the PZZ mixed‐matrix membranes (Figure 2A). The PZO copolymer displayed a predominantly amorphous profile, arising from the nine ethylene oxide units in POEM, which are insufficient to form a regular crystalline structure. In contrast, a weak diffraction peak at 6.5° corresponds to the crystalline ordering of ionic PZA chains, which imparts enhanced mechanical strength [36, 37]. Notably, this PZA peak diminished at 5 wt.% NZIF‐8 loading and disappeared entirely above 10 wt.%. This indicates a disruption of PZA crystallinity upon the incorporation of NZIF‐8, suggesting that Zn^2+^ ions in PZO preferentially interact with ZIF‐8 ligands rather than maintaining coordination with their original acrylate groups. Such redistribution of Zn^2+^ coordination may enhance CO_2_ transport by increasing the availability of active complexation sites. Furthermore, the characteristic (011) reflection of ZIF‐8 at 7.44°, associated with its six‐membered ring aperture, significantly shifted to 7.30° in the PZZ membranes (Figure 2B). This shift implies a structural perturbation of the ZIF framework, likely arising from strong polymer‐filler interactions, including the partial infiltration of PZO chains into the ZIF‐8 apertures [38, 39]. The peak shift was also observed in the PZZ membrane with IZIF‐8, indicating that the peak shift is independent of the filler morphology (Figure S15).

To confirm that this (011) peak shift is unique to the PZO matrix, TFC‐MMMs were prepared by embedding NZIF‐8 into four additional polymer matrices under identical conditions: 6FDA‐DAM, PDMS, PTMSP, and homopolymerized POEM, P(POEM) (Figure 2C). 6FDA‐DAM is a rigid, fluorinated polyimide; PDMS is a flexible silicone elastomer; PTMSP is a glassy, high‐permeability polysiloxane; and P(POEM) is the non‐crosslinked homopolymer component of PZO. By comparing these five MMMs, the specific effect of the Zn^2+^‐acrylate sites in PZO can be isolated. The resulting XRD patterns (Figure 2D,E) show that the characteristic ZIF‐8 (011) reflection at 7.44° remains unchanged in the 6FDA‐DAM, PDMS, PTMSP, and P(POEM) membranes, indicating no lattice contraction. In stark contrast, only the PZZ MMM exhibits the pronounced shift to 7.30°, confirming that the pore tuning is uniquely dependent on the PZO matrix. This unique peak shift is attributed to the Zn^2+^ in PZO: the divalent ions coordinate with imidazolate linkers at the cage apertures, anchoring the ZIF‐8 framework and drawing them slightly inward. Such metal‐ligand interactions rigidify the surface pore windows and reduce the effective aperture size.

#### 2.2.2 Validation of Strong Polymer‐Filler Interaction

FT‐IR analysis was conducted to investigate chemical interactions between the PZO copolymer and NZIF‐8 (Figure 2F). The asymmetric stretching bands of the zinc carboxylate group in PZO appeared at 1599 and 1558 cm^−1^ [40]. Upon incorporation of NZIF‐8, these bands blue‐shifted to 1604 and 1578 cm^−1^, respectively, consistent with strong electrostatic interactions between the PZA carboxylate groups and ZIF‐8 ligands. In contrast, the C─O─C ether stretching band of the POEM segment showed only a minor shift from ∼1100 to 1096 cm^−1^, suggesting weaker interactions. This indicates that despite the inherent polarity of the ethylene oxide groups, the interaction between zinc acrylate units and NZIF‐8 is dominant within the polymer matrix. XPS analysis further confirmed these interactions (Figure 2G). The Zn 2p binding energy in PZO progressively shifted with increasing NZIF‐8 content, supporting a strong polymer‐filler interaction that alters the coordination environment of the Zn ions in the PZO copolymer [41]. DSC measurements (Figure 2H) showed that the glass transition temperature (T_g_) of the POEM segments steadily increased from −63.7°C to −60.3°C with NZIF‐8 loading, reflecting restricted chain mobility [42]. Although POEM itself exhibits weak direct interaction with ZIF‐8, the strong anchoring of PZA units likely suppresses the motion of the entire polymer network through physical confinement.

However, excessive polymer‐filler interaction can lead to pore blockage, adversely affecting gas diffusion. To assess whether the electrostatic interaction between PZO and NZIF‐8 creates a favorable interface without obstructing transport pathways, we conducted a comparative study. NZIF‐8 was mixed with either the PZO copolymer or neat P(POEM), followed by rigorous washing (≥3 cycles) to remove any non‐bound, surface‐adsorbed polymer. This procedure was designed to test whether strong interfacial compatibility would result in persistent polymer retention, altering the pore structure even after washing. Nitrogen adsorption‐desorption isotherms revealed a significant decrease in BET surface area for PZO‐treated NZIF‐8 (PZO@NZIF‐8) from 2244 to 1036 m^2^/g, whereas P(POEM)‐treated NZIF‐8 showed only a modest reduction (Figure S16). This confirms the much stronger binding of PZO to NZIF‐8, attributed to the Zn^2+^‐ligand interactions absent in P(POEM). Importantly, while the average pore size (11.6 Å) in PZO@NZIF‐8 remained nearly unchanged, the reduced signal intensity in the pore size distribution suggests partial infiltration rather than full pore blockage. Furthermore, XRD patterns of PZO@NZIF‐8 exhibited the same (011) peak shift to 7.30° observed in the PZZ membranes (Figure S17), confirming that PZO chains uniquely penetrate and anchor within the framework. Collectively, these results demonstrate that Zn^2+^‐acrylate sites in PZO create a durable, well‐integrated polymer‐MOF interface that enables effective pore tuning without completely compromising the porous network, thereby preserving pathways for gas transport.

### 2.3 Gas Separation Performance of PZZ TFC MMMs

#### 2.3.1 Gas Transport and Selectivity Under Single‐ and Mixed‐Gas Conditions

**FIGURE 3:**
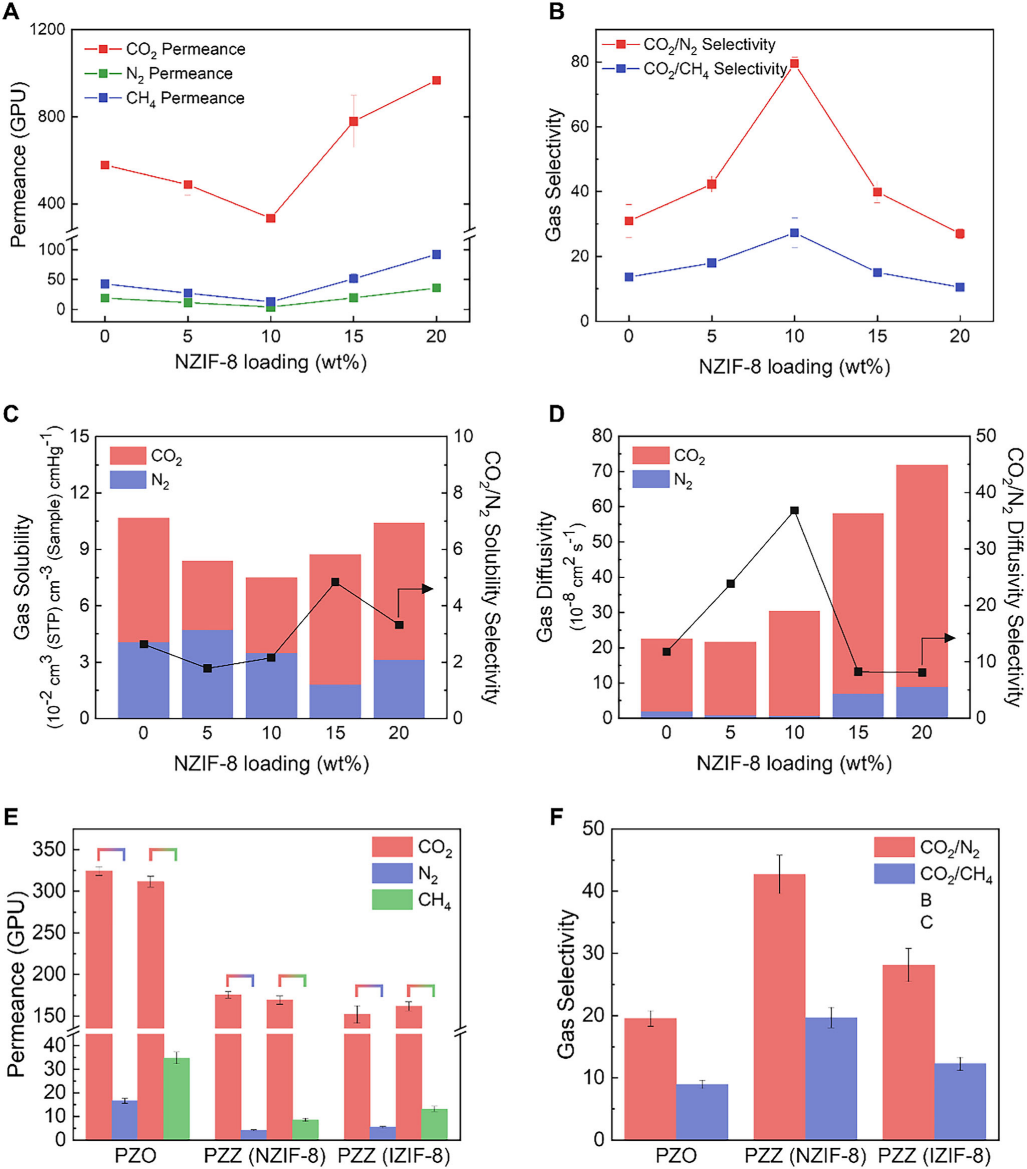
Gas separation performances of PZZ TFC MMMs. (A) Single‐gas CO_2_, N_2_, and CH_4_ permeance of PZZ membranes. (B) Ideal CO_2_/N_2_, CO_2_/CH_4_ selectivity of PZZ membranes. (C) CO_2_ and N_2_ solubility, CO_2_/N_2_ solubility selectivity of PZZ membranes as a function of NZIF‐8 loading. (D) CO_2_ and N_2_ diffusivity, CO_2_/N_2_ diffusivity selectivity of PZZ membranes as a function of NZIF‐8 loading. (E) CO_2_, N_2_, and CH_4_ permeance of PZZ membranes measured with equimolar CO_2_/N_2_ and CO_2_/CH_4_ mixture. (F) CO_2_/N_2_, CO_2_/CH_4_ selectivity of PZZ membranes.

The CO_2_ separation performance of PZZ membranes was evaluated at 1 atm and 25°C (Figure 3A,B; Table S2). The neat PZO copolymer exhibited a CO_2_ permeance of 580 GPU, with ideal CO_2_/N_2_ and CO_2_/CH_4_ selectivities of 31 and 14, respectively. Despite the high content of ZA, PZO demonstrated robust separation, likely via Zn^2+^‐facilitated CO_2_ transport [43, 44]. As NZIF‐8 loading was increased to 10 wt.% (denoted as PZZ‐10), CO_2_ permeance gradually decreased, reflecting partial pore infiltration by PZO chains and an increased selective‐layer thickness. In contrast, selectivity for both CO_2_/N_2_ and CO_2_/CH_4_ more than doubled, reaching 80 and 27, respectively. These results confirm that NZIF‐8 provides an effective size‐sieving effect. Although ZIF‐8's gate‐opening can expand its window to 4.0 Å, PZO‐ligand interactions immobilize the imidazole linkers, restoring an effective aperture near the intrinsic 3.4 Å size and thereby hindering N_2_ and CH_4_ permeation. The consistent performances across different samples that were cut from random positions confirmed the uniformity of the large‐area membranes (Figure S18). To validate the role of Zn^2+^‐acrylate functionality, a control membrane was fabricated using a neat P(POEM) homopolymer (Figure S19). This NZIF‐8/P(POEM) membrane displayed a dramatic decrease in selectivity, with CO_2_/N_2_ and CO_2_/CH_4_ values dropping to 41 and 15. This marked decrease demonstrates that the ionic Zn^2+^ sites in PZA are essential not only for effective pore tuning but also for enhancing gas separation through strong electrostatic interactions.

To further investigate the gas transport mechanism in PZZ membranes, solubility and diffusivity were calculated from equilibrium sorption isotherms based on the solution‐diffusion model (Figure 3C,D; Figure S20) [45]. The highly increased CO_2_/N_2_ selectivity in the PZZ‐10 membrane is predominantly attributed to a massive increase in diffusivity selectivity [46, 47, 48, 49]. While solubility selectivity remained nearly constant across all NZIF‐8 loadings, indicating negligible change in sorption, the diffusivity selectivity surged from 12 to 37. This confirms that the high‐aspect‐ratio NZIF‐8 establishes size‐selective channels that preferentially accelerate CO_2_ diffusion over N_2_, owing to CO_2_’s smaller kinetic diameter. Consequently, the enhancement in overall CO_2_/N_2_ separation is overwhelmingly driven by diffusivity, underscoring the critical role of NZIF‐8's engineered physical structure in achieving effective membrane selectivity.

#### 2.3.2 Morphology‐Driven Enhancement in NZIF‐8

To investigate the effect of particle morphology on gas separation, we fabricated TFC MMMs using IZIF‐8, a filler with a conventional rhombic dodecahedron shape (Figure S21). The IZIF‐8‐based MMMs exhibited a CO_2_ permeance trend similar to the PZZ (NZIF‐8) membranes: permeance initially decreased with filler loading before increasing at higher concentrations. The optimal performance for IZIF‐8 MMMs was achieved at 15 wt.% loading, delivering a CO_2_ permeance of 371 GPU with CO_2_/N_2_ and CO_2_/CH_4_ selectivities of 53 and 21, respectively. While its CO_2_ permeance was comparable to the PZZ‐10 membrane, its gas selectivities were substantially lower. This result underscores that the sheet‐like morphology of NZIF‐8 significantly enhances separation performance. Although both filler types permit in‐plane CO_2_ permeation, the high‐aspect‐ratio NZIF‐8 forces N_2_ and CH_4_ molecules to follow longer, more tortuous pathways [50, 51]. This extended diffusion path hinders the transport of these larger gases, thereby maximizing the molecular sieving effect. At loadings above the optimum (e.g., 20 wt.%), particle aggregation introduces non‐selective cracks and defects, leading to increased permeance but decreased selectivity.

The mixed‐gas separation performance was then evaluated using an equimolar CO_2_/N_2_ and CO_2_/CH_4_ mixture at 1 atm and 25°C (Figure 3E,F). Under these conditions, performance was slightly lower than in single‐gas tests, a drop attributed to competitive sorption between gas molecules and the plasticization effect of CO_2_ [52, 53, 54]. Despite this, the PZZ‐10 membrane retained a respectable CO_2_ permeance of 176 GPU, with CO_2_/N_2_ and CO_2_/CH_4_ selectivities of 43 and 20. Consistent with the single‐gas results, the PZZ‐10 membrane still outperformed its IZIF‐8 counterpart. Specifically, the CO_2_/N_2_ and CO_2_/CH_4_ selectivities of the PZZ‐10 membrane were 117% and 130% higher, respectively, than those of the IZIF‐8 membrane, reaffirming the significant morphological advantage of NZIF‐8.

To further leverage the benefits of NZIF‐8 and boost permeance, we fabricated an ultrathin PZZ‐10 membrane (denoted thin PZZ‐10) by reducing the casting solution concentration. This yielded a selective layer with a thickness of 158 nm (Figure S22), causing the CO_2_ permeance to surge to nearly 1000 GPU. However, this came at the cost of selectivity, with the CO_2_/N_2_ and CO_2_/CH_4_ values decreasing to 41.2 and 15.2, respectively. Despite this trade‐off, the performance remains well within the commercially attractive region for post‐combustion CO_2_ capture. This decline in selectivity is likely due to the formation of non‐selective defects, as the layer thickness is similar to or even smaller than the lateral dimensions of the NZIF‐8s. Therefore, we hypothesize that precise control over the thickness and orientation of NZIF‐8 will be crucial for preserving high selectivity in ultra‐thin TFC membranes.

#### 2.3.3 Mixed‐Gas Performance Under Various Conditions and Mechanism Analysis

**FIGURE 4:**
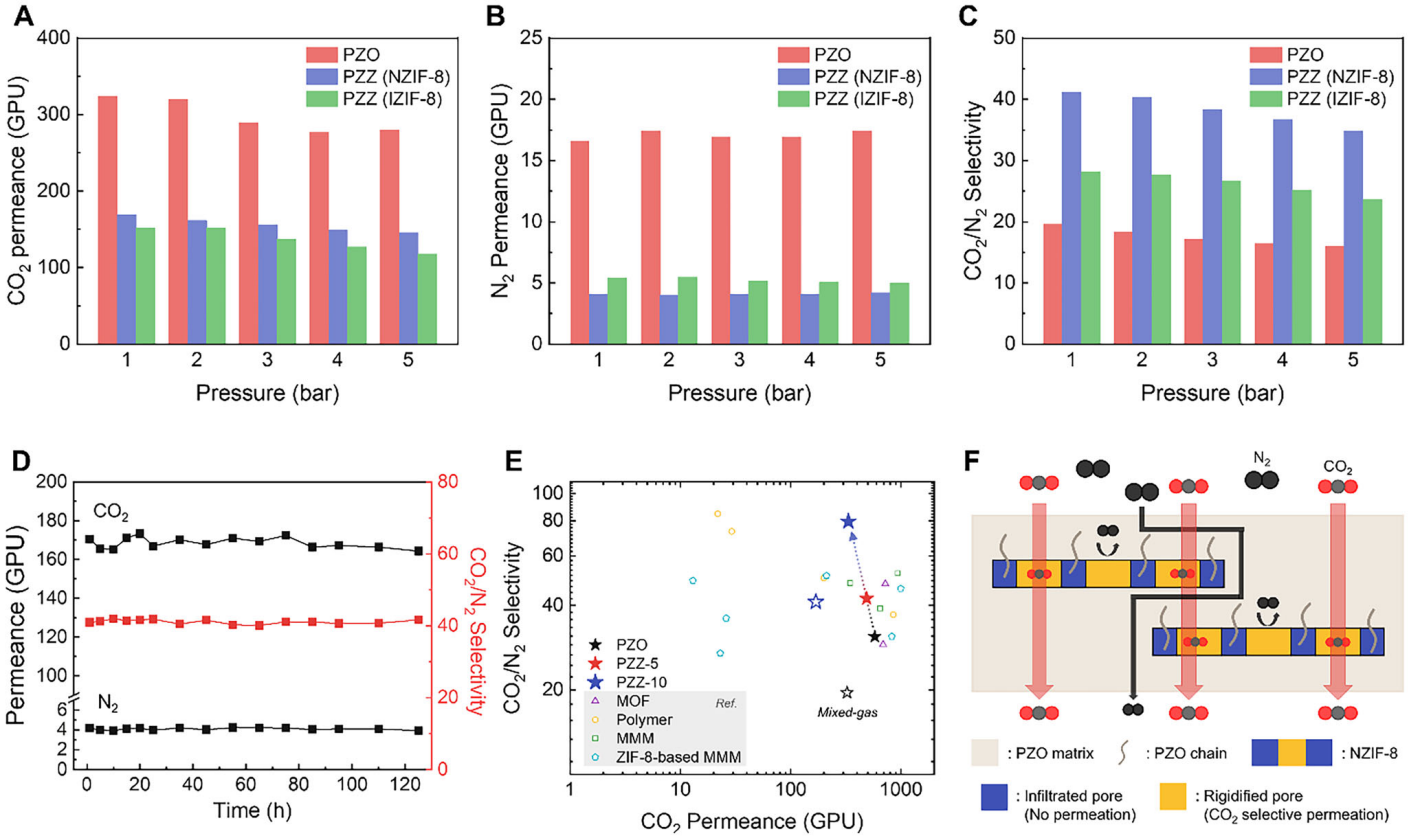
Gas separation performances of PZZ‐10 membranes depending on various conditions. (A–C) Mixed gas separation performance of PZO and PZZ‐10 membranes depending on feed pressure. (A and B) Permeance of CO_2_ and N_2_. (C) CO_2_/N_2_ selectivity. (D) Long‐term CO_2_/N_2_ separation test for 125 h. All mixed gas separation tests were conducted at 1 bar, 25°C with an equimolar mixture. (E) The plot of CO_2_ permeance versus CO_2_/N_2_ selectivity of the PZZ membranes and comparison with previously reported literature. (F) Gas transport phenomena of pore‐tuned NZIF‐8 with high aspect ratio in TFC MMM with rigidified pores.

The influence of feed pressure on single‐gas separation was evaluated for the PZZ‐10 membrane at 1 and 5 bar (Figure S23). When the pressure was increased to 5 bar, CO_2_/N_2_ selectivity decreased from 80 to 57, and CO_2_/CH_4_ selectivity decreased from 27 to 19. Conversely, CO_2_ permeance rose slightly from 333 to 350 GPU. This behavior suggests that the higher pressure partially relaxes the linker constraints within the ZIF‐8 structure, attenuating the suppression of ligand movement and thus enlarging the effective pore aperture. Consequently, the size‐sieving property of NZIF‐8 is diminished under high‐pressure conditions. We then examined the mixed‐gas performance using an equimolar CO_2_/N_2_ mixture at sequential pressures from 1 to 5 bar (Figure 4A–C). In all cases, both permeance and selectivity declined with increasing pressure. Nevertheless, the PZZ‐10 membrane consistently outperformed an IZIF‐8–based MMM under identical conditions, confirming that NZIF‐8's nanoplate morphology more effectively sustains separation performance under pressure than isotropic particles.

In addition, long‐term stability was assessed using the best‐performing PZZ‐10 membrane (Figure 4D). The membrane exhibited no significant performance degradation over 125 h of continuous operation, retaining a CO_2_ permeance above 164 GPU and CO_2_/N_2_ selectivity over 40. Since practical post‐combustion flue gas contains significant water vapor, the membrane's performance was also tested under humid conditions for 50 h (Figure S24). Initially, CO_2_ permeance and CO_2_/N_2_ selectivity decreased slightly compared to performance under dry conditions, likely due to competitive permeation from water molecules. After 24 h, gas permeance began to increase gradually, which can be attributed to the swelling of polymer chains upon water absorption. Notably, only a 7% drop in CO_2_/N_2_ selectivity was observed after 50 h, indicating the membrane's robustness and applicability for humid gas separations. Furthermore, extended long‐term evaluations using a simulated flue gas mixture (15 vol% CO_2_ / 85 vol% N_2_) confirmed the absence of plasticization and sustained stability under both dry and humid conditions (Figures S25 and S26), reinforcing the membrane's suitability for industrial applications.

The gas separation performance of the PZZ membranes was benchmarked against various state‐of‐the‐art membranes (Figure 4E; Table S3) [23, 31, 32, 55, 56, 57, 58, 59, 60, 61, 62, 63, 64, 65, 66], demonstrating excellent CO_2_ selectivity among MOF‐based MMMs. While high permeability values are often reported in literature, achieving this performance in practical, defect‐free thin‐film membranes remains a significant challenge. Our work successfully bridges this gap by pioneering a “polymer‐in‐cage” strategy, where the matrix polymer itself acts as an active agent to remodel the filler's pore environment and enhance interfacial compatibility. This exceptional performance arises from three synergistic factors originating from this integrated design. First, the Zn^2+^‐acrylate sites in PZO electrostatically anchor and rigidify the ZIF‐8 pore windows near 3.4 Å. Second, the high‐aspect‐ratio NZIF‐8s create long, tortuous in‐plane pathways that maximize diffusivity selectivity. Third, Zn^2+^‐facilitated transport further enhances CO_2_ permeation (Figure 4F). The successful fabrication of this high‐performance coating is enabled by the unique filler morphology and strong interfacial interactions; the nanosheets help minimize defect formation, while enhanced compatibility between the filler and polymer suppresses non‐selective channels. Furthermore, the stable adhesion between the PZO polymer and the porous support contributes to the formation of a uniform, submicron selective layer.

Together, these findings establish a versatile polymer‐in‐cage strategy for MOF interface engineering and pore tuning. Future work will focus on reducing the thickness of both the NZIF‐8 and the overall selective layer to further boost permeance without sacrificing selectivity, paving the way for next‐generation high‐performance gas separation membranes.

## 3 Conclusions

In conclusion, this work establishes a versatile “polymer‐in‐cage” strategy, demonstrating that a zinc‐acrylate‐based copolymer can function dually as a mechanically robust matrix and as an active pore‐modulating agent for high‐aspect‐ratio NZIF‐8s. Through a simple solution‐blending process, strong electrostatic interactions between the copolymer's Zn^2+^ sites and the ZIF‐8 imidazolate linkers induce partial chain infiltration into the framework's six‐membered windows. These interactions effectively constrain linker mobility, narrowing the pore aperture from its flexible, swing‐expanded state (∼4.0 Å) to its intrinsic size (∼3.4 Å) and thereby enhancing the molecular sieving of CO_2_ over N_2_ and CH_4_. At an optimal 10 wt.% NZIF‐8 loading (PZZ‐10), the resulting membranes exhibited a CO_2_ permeance of 333 GPU with CO_2_/N_2_ and CO_2_/CH_4_ selectivities of 80 and 27, respectively. This performance surpasses that of both the neat PZO polymer and controls using isotropic ZIF‐8. The membranes also demonstrated excellent operational durability, maintaining stable performance for over 125 h under mixed‐gas feeds up to 5 bar and in humid conditions (85% RH). These achievements highlight the power of a synergistic design that integrates filler morphology with polymer‐filler interface engineering. The anisotropic nanoplates create extended, tortuous pathways that amplify diffusivity selectivity, while the Zn^2+^ moieties contribute to facilitated CO_2_ transport. Looking ahead, precisely controlling the nanoplate thickness and further refining the ultra‐thin selective‐layer casting process will be key to boosting permeance without sacrificing the high selectivity achieved here. By uniquely marrying polymer chemistry with MOF architecture in a single, scalable step, this strategy paves the way for the next generation of high‐performance membranes for CO_2_ capture and other critical separations.

## 4 Experimental Section/Methods

### 4.1 Synthesis of ZIF‐8 Nanoplates (NZIF‐8)

Zn_5_(NO_3_)_2_(OH)_8_ (Zinc layered hydroxide) nanosheets were first synthesized as a precursor. Zn(NO_3_)_2_·6H_2_O (5.9498 g, 0.02 mol) and LiOH·H_2_O (0.4198 g, 0.01 mol) were dissolved in 100 and 50 mL of DI water, respectively. The prepared lithium hydroxide solution was slowly injected into the zinc nitrate solution for 4 h with a syringe pump. The mixed solution was additionally stirred for 4 h. The white precipitates were collected on a polyethersulfone substrate (0.03 µm pore diameter, GVS, circle type, 47 mm diameter) by vacuum filtration. They were further washed several times with DI water for purification and dried at room temperature in a vacuum oven overnight. 0.12 g of as‐prepared precursor nanosheets and 2‐methylimidazole (1.8 g, 0.02 mol) were dispersed in 50 and 100 mL of acetone, respectively. The prepared 2‐methylimidazole solution was slowly injected into the zinc‐layered hydroxide nanosheet solution for 12 h with a syringe pump and additionally stirred for 20 h. The white precipitates were collected on a nylon substrate (0.1 µm pore diameter, Sterlitech, circle type, 47 mm diameter) by vacuum filtration. They were further washed with acetone several times for purification and dried at room temperature in a vacuum oven overnight to remove residual solvents.

### 4.2 Synthesis of PZA‐co‐POEM Copolymer

The PZA‐co‐POEM comb‐shaped copolymer (referred to as PZO) was synthesized through a commercially available free‐radical polymerization method. Initially, 2 g of ZA was added to 40 m of DMSO in a round‐bottom flask. The flask was stirred at 60°C for 2 h to completely dissolve the ZA. After the flask was cooled down to room temperature, 8 g of POEM was added to the flask. Subsequently, 0.01 g of AIBN was introduced into the solution, and the mixture was subjected to 30 min of N_2_ purging. The flask was then immersed in an oil bath at 70°C for 18 h to facilitate polymerization. After the polymerization, the resulting material was precipitated using an excess of n‐hexane and was washed with n‐hexane three times to eliminate any remaining monomers. Then, the resultant was dried in an oven at 70°C overnight to remove the residual solvent. For comparison with PZO copolymer, homopolymer P(POEM) was synthesized using the same procedure, with 10 g of POEM without ZA.

### 4.3 Fabrication of the TFC MMMs

To prepare the solution for the mixed‐matrix membrane, the PZO copolymer was first dissolved in a mixed solvent of EtOH and DI water in a volumetric ratio of 7:3 at 50°C for 3 h. Subsequently, a tailored quantity of ZIF‐8s was incorporated into the PZO copolymer solution with stirring at 50°C for 3 h and at room temperature for 24 h. The composite membranes were prepared using a solution‐casting method. First, a PTMSP gutter layer was applied onto the polysulfone porous support. A solution of 1.5 wt.% PTMSP in cyclohexane was cast on the support membrane using an RK Control coater (Model 101, Control RK Print‐Coat Instruments Ltd., UK), and it was allowed to air‐dry at room temperature. Concurrently, the PZO/NZIF‐8 solution was cast onto the PTMSP‐coated polysulfone support. The membrane was dried at 50°C for 24 h and at 90°C for 2 h for the complete removal of the solvent. The amount of NZIF‐8 was varied at 5, 10, 15, and 20 wt.% of the PZO copolymer, respectively. The resulting composite membranes were designated as PZZ‐X, where X corresponds to the weight percentage of NZIF‐8 compared to PZO copolymer.

## Conflicts of Interest

The authors declare no conflicts of interest.

## Supporting information




**Supporting File**: advs74473‐sup‐0001‐SuppMat.docx.

## Data Availability

The data that support the findings of this study are available from the corresponding author upon reasonable request.
