# Supplementary material for: Polymer‐in‐Cage Strategy for Pore Tuning of High‐Aspect Ratio ZIF Nanoplate: Toward Sub‐Micrometer‐Thick Large Area CO2 Separation Membranes
Source: Adv Sci (Weinh). 2026 Feb 16;13(28):e19351. doi: 10.1002/advs.202519351 (PMC13185815; doi:10.1002/advs.202519351)
Supplement: Supplementary file 1 — Supporting File: advs74473‐sup‐0001‐SuppMat.docx. [file ADVS-13-e19351-s001.docx]

Supporting Information

Polymer-in-Cage Strategy for Pore Tuning of High-Aspect Ratio ZIF Nanoplate: Towards Sub-micrometer-thick Large Area CO_2_ Separation Membranes

Minsu Kim^†^, Hyo Jun Min^†^, Min Kyu Choi, Ki Chul Kim, Bomi Kim, Miso Kang, Nahyeon Lee, Kiwon Eum, Jong Hak Kim*, Dae Woo Kim*

† These authors contributed equally to this work.

Extended Experimental Section/Methods

*Materials*: Zinc acrylate (ZA), poly (oxyethylene methacrylate) (POEM, poly(ethylene glycol) methyl ether methacrylate, Mn = 500 g mol^−1^), and were purchased from Sigma-Aldrich. 2,2′-Azobis(2-methylpropionitrile) (AIBN, 98%) was obtained from Acros Organic. Poly(1-(trimehthylsilyl-1-propyne)) (PTMSP) was purchased from Gelest. Zn(NO_3_)_2_·6H_2_O (Zinc nitrate hexahydrate, 98%), LiOH·H_2_O (Lithium hydroxide monohydrate, ≥98%), and 2-methylimidazole (CH_3_C_3_H_2_N_2_H, 99.0%) were purchased from Sigma-Aldrich for the synthesis of ZIF-8 particles. Acetone (CH_3_COCH_3_, extra pure), methanol (CH_3_OH, GR), deionized (DI) water, ethanol (EtOH), n-hexane, and dimethylsulfoxide (DMSO) were purchased from DUKSAN Industry Inc. A microporous membrane made of polysulfone on non-woven fabric for support was provided by Toray Chemical Korea Inc. All the chemical materials and solvents were utilized as received without any modification or purification.

*Synthesis of isotropic ZIF-8 (IZIF-8) particles*: 1.4 g of Zn(NO_3_)_2_·6H_2_O and 2-methylimidazole were dissolved in 100 mL of methanol separately. Two solutions were directly mixed in a separate flask and stirred for an hour. The white precipitates were centrifuged and washed with methanol several times. The collected precipitates were dried at room temperature in a vacuum oven overnight to remove residual methanol.

*Computational methods*: Molecular dynamics (MD) simulations were conducted to investigate the effect of the PZO copolymer on pore tuning in ZIF-8 nanoplates and its influence on molecular sieving behaviors. Two simulation models were designed depending on the absence or presence of copolymer chains: (i) a pristine ZIF-8 surface model and (ii) model of ZIF-8 surface with partially infiltrated PZO copolymer chains. Each simulation model was equilibrated under the NVT ensemble at 298 K and then stabilized under the NPT ensemble at 298 K and 1 atm for 1 ns. To quantitatively assess the impact of PZO copolymer infiltration on the pore structure of ZIF-8 system, three H-H distance pairs in six-membered rings at three distinct pore sites were measured over simulation time. The distinct pore sites are classified as (i) penetration pore, through which PZO copolymer was directly infiltrated into the ZIF-8 surfaces, (ii) surface pore neighboring in horizontal direction relative to the penetration pore, and (iii) subsurface pore neighboring in perpendicular direction relative to the penetration pore. This allowed us to conduct a comparative analysis between pristine ZIF-8 surface and its derivative embedded by the partial infiltration of PZO copolymer chains in terms of the extent of pore contraction. All simulations were performed using the Large-scale Atomic/Molecular Massively Parallel simulation package, with the DREIDING force field applied to both the polymer and the ZIF-8 framework.1-2 Electrostatic interactions were incorporated by computing atomic charges using the GAUSSIAN 16 program with the B3LYP functional and the 6-311+G(d,p) basis set.3-4

*Gas separation measurements*: Gas adsorption isotherms of CO_2_ and N_2_ were measured using a pressure decay method. The gas uptake measurements were obtained by monitoring the decrease in pressure within the fixed-volume chamber. Experiments were conducted using 0.15 g of samples, which underwent thorough degassing at 120℃ under vacuum for at least 12 h and stabilized at 35℃ before experiments. The adsorption capacity was fitted with the Langmuir model, considering that the membranes possess a finite number of adsorption sites.

Single gas permeance of the membrane was assessed using a constant pressure/variable volume apparatus (Airrane Co. Ltd. (Korea)) under conditions of 1 bar, 25℃. The active area of each membrane was 10.2 cm^2^. Pure gas permeance is presented in GPU (1 GPU = 10^−6^ cm^3^(STP)/(s·cm^2^·cmHg)), and the gas selectivity of the membranes was calculated by dividing the permeance of the target gas from another.

Mixed gas permeation tests were performed using the Wicke–Kallenbach technique under conditions of 1 bar, 25℃. Equimolar gas mixtures of CO_2_/N_2_ and CO_2_/CH_4_ were used. The argon sweep gas also flowed at 40 mL/min, while the component gases flowed at 20 mL/min, respectively. The composition of the permeate gas on the sweep side of the membranes was analyzed by gas chromatography (YL 6500 GC, YoungIn Chromass). Before the gas concentration on the permeate side was measured by gas chromatography, a minimum equilibration time of 1 h was applied.

*Characterizations*: The synthesis of the copolymer and the chemical interaction between the copolymer and the NZIF-8 were investigated by FT-IR spectrometer (Spectrum Two, Perkin Elmer, USA). The chemical compositions of PZO copolymer were calculated by 400 MHz 1H-NMR spectrometer (AVANCE III HD 400, Bruker, Germany) using D_2_O as a solvent. XRD analysis was conducted at a scanning speed of 2°/min in the 2θ range of 5°-50° (SmartLab, Rigaku, Japan). Differential scanning calorimetry (DSC; Discovery DSC, TA Instruments, USA) was applied at a heating rate of 20℃ min^−1^ under N_2_ atmosphere. SEM (JSM-7001F, JSM-7610F-Plus, JEOL Ltd., Japan) images were obtained to characterize the NZIF-8 and PZZ thin-film composite membranes. The thickness of the membranes was obtained from cross-sectional SEM images of the membranes. Specific surface area and the pore-size distribution using the non-localized density functional theory (NLDFT) of various ZIF-8 particles were obtained by N_2_ adsorption-desorption isotherms using a gas adsorption instrument (Autosorb IQ, Quantachrome, USA).


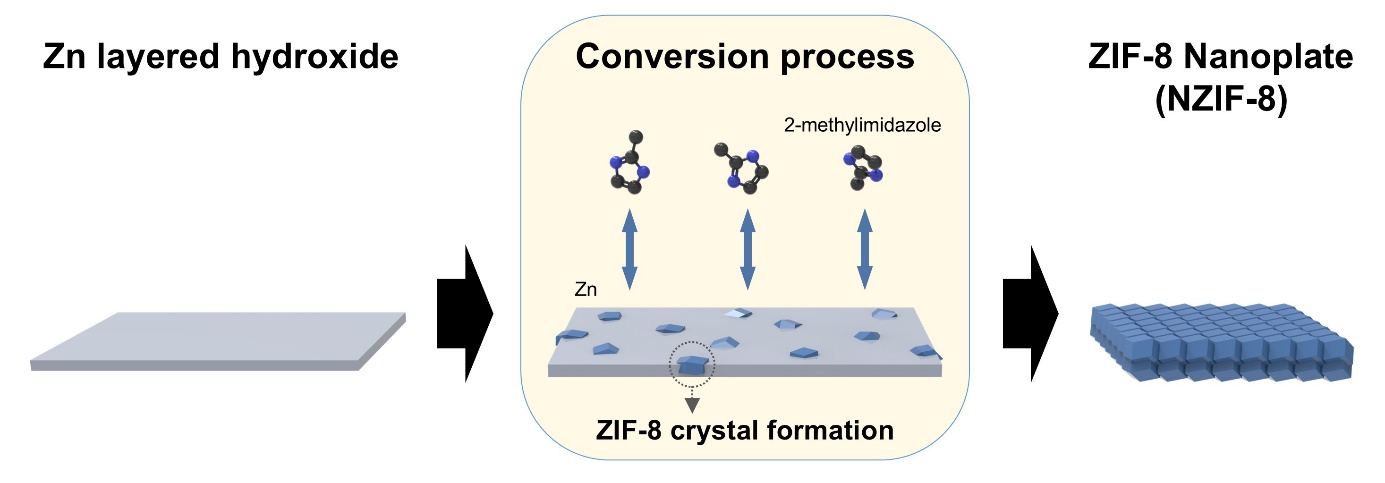


Figure S1. Schematic illustration of NZIF-8 synthesis *via* conversion method.


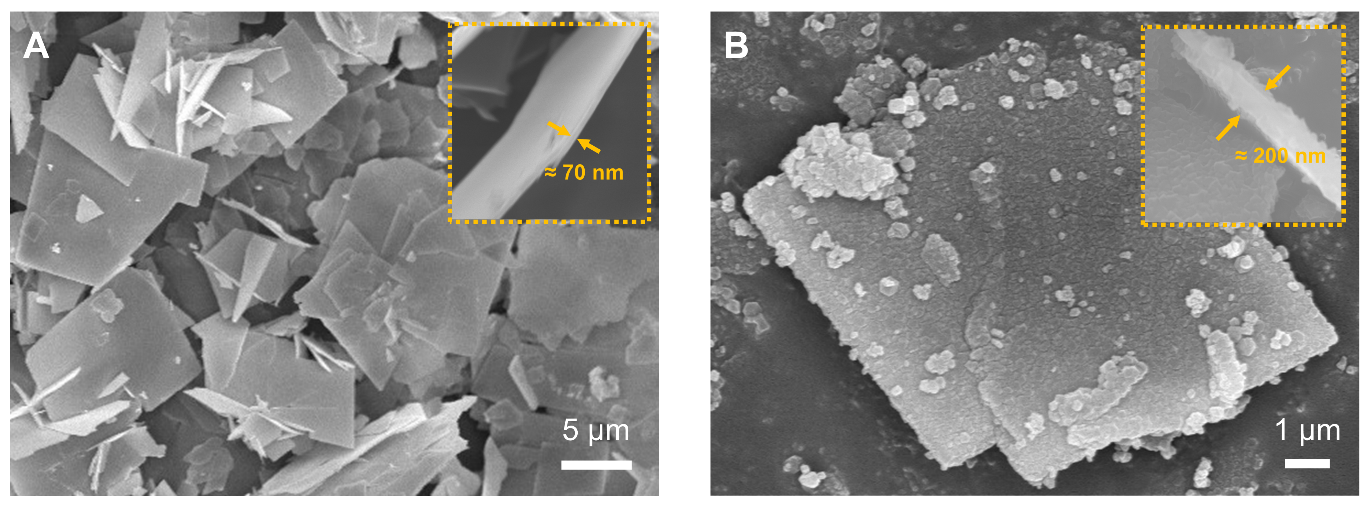


Figure S2. A, SEM image of synthesized Zn layered hydroxide. B, SEM image of synthesized NZIF-8. Insets are higher magnification images representing approximate thickness of the particles.


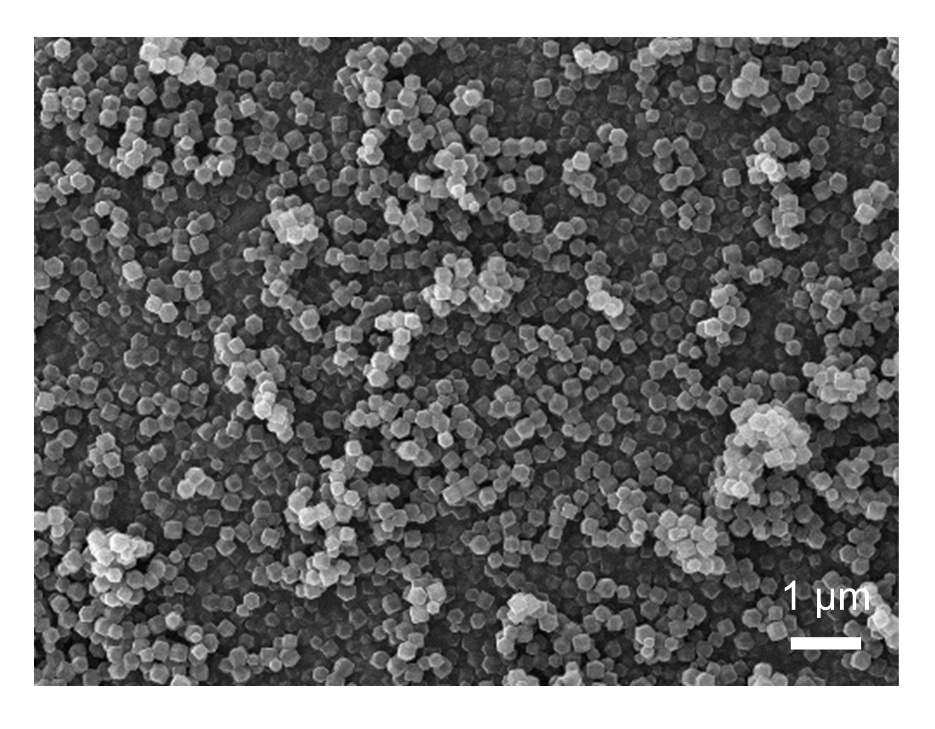


Figure S3. SEM image of IZIF-8.


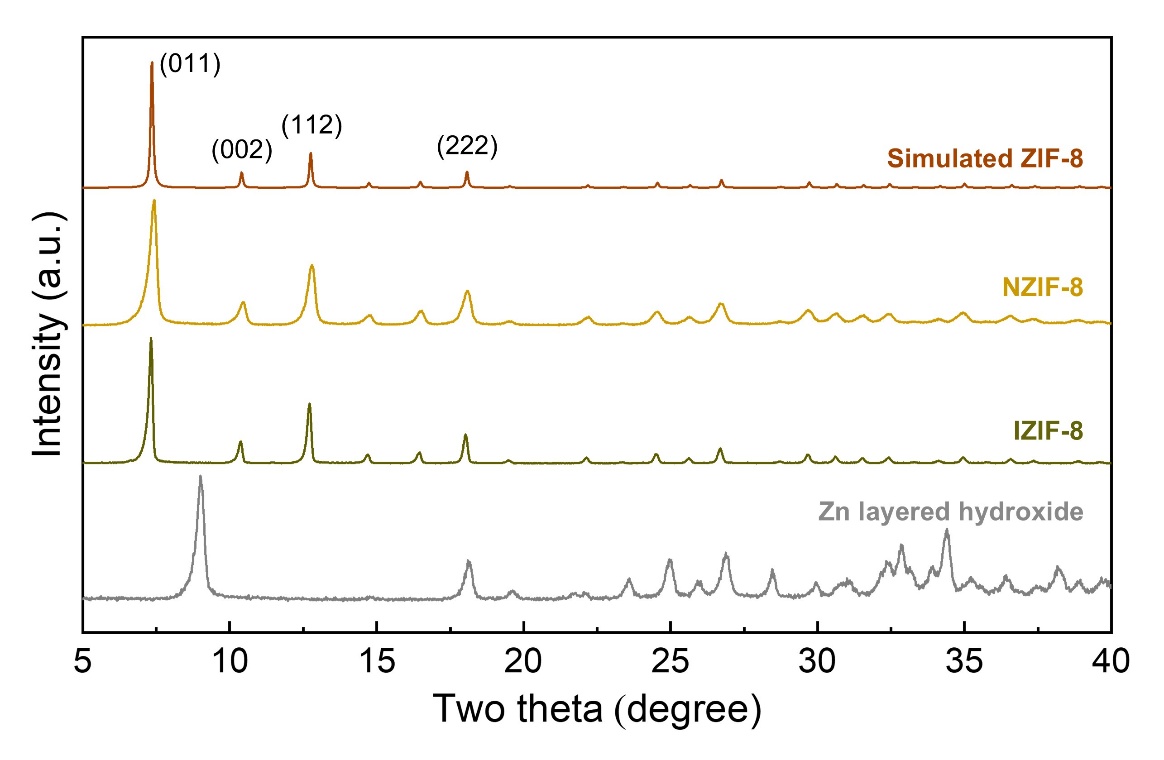


Figure S4. XRD patterns of the synthesized particles.


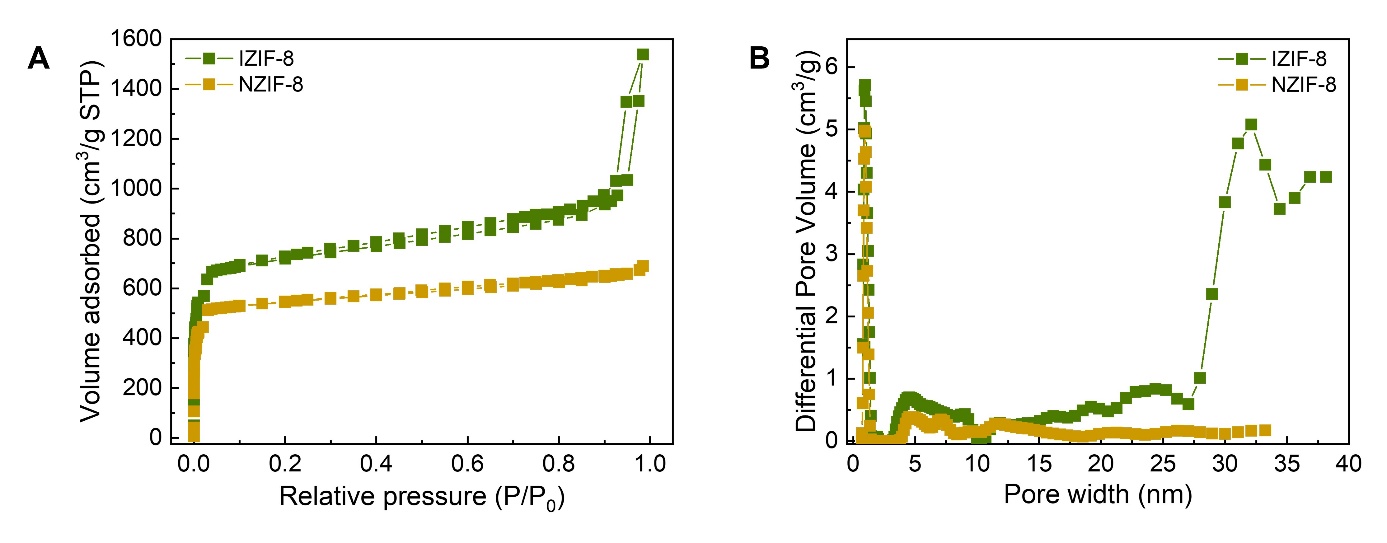


Figure S5. A, N_2_ adsorption-desorption isotherms and B, pore size distribution of ZIF-8s measured at 77K. STP, Standard temperature and pressure.


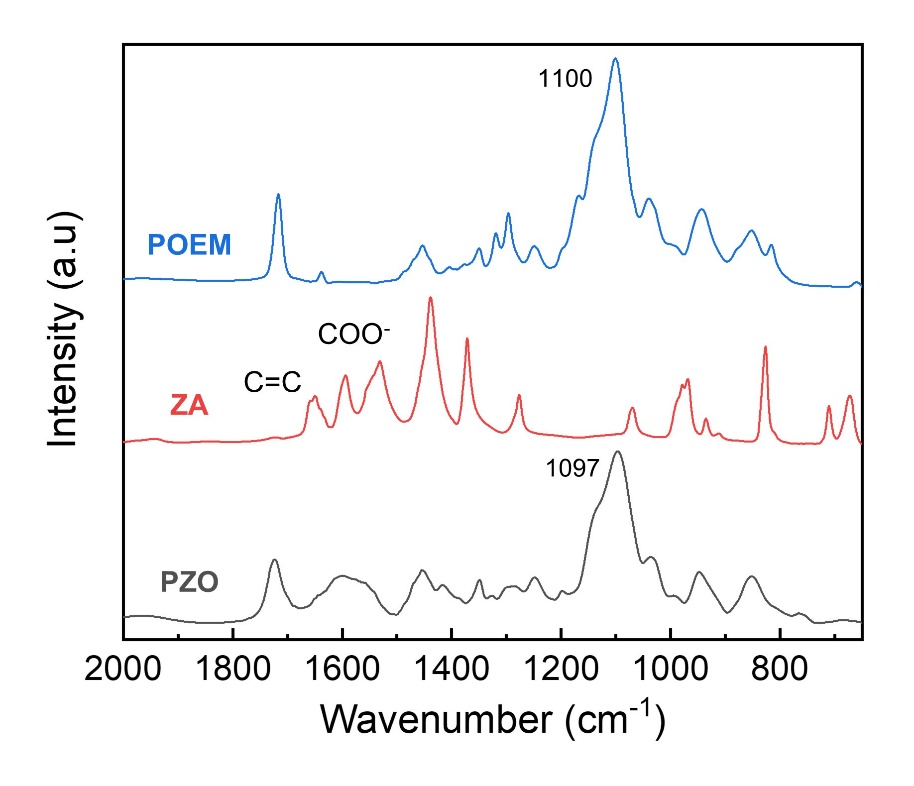


Figure S6. FT-IR spectra of POEM, ZA monomers and synthesized PZO copolymer.


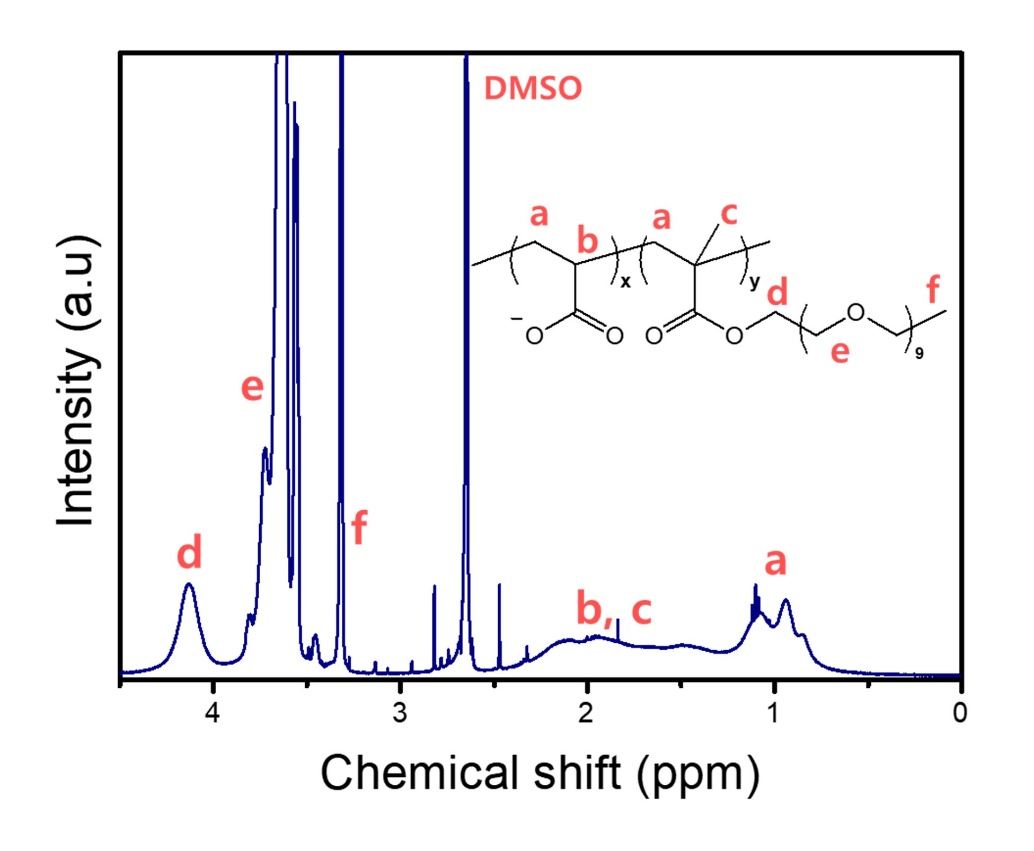


Figure S7. ^1^H-NMR spectrum of PZO copolymer.


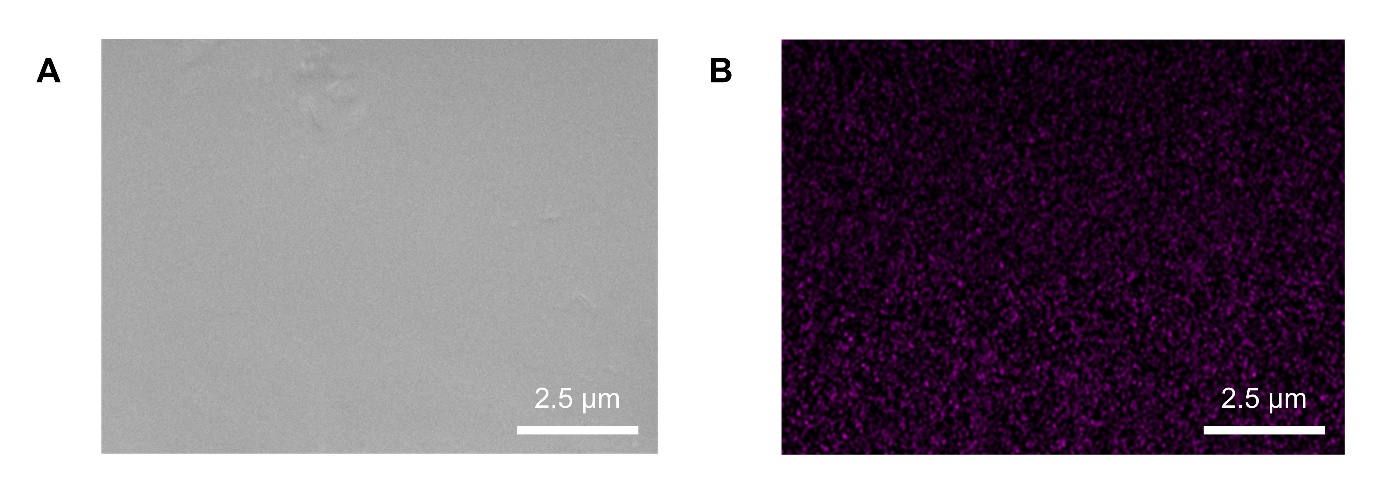


Figure S8. A, Surface SEM image of PZO and B, EDS elemental mapping of Zn in PZO copolymer.


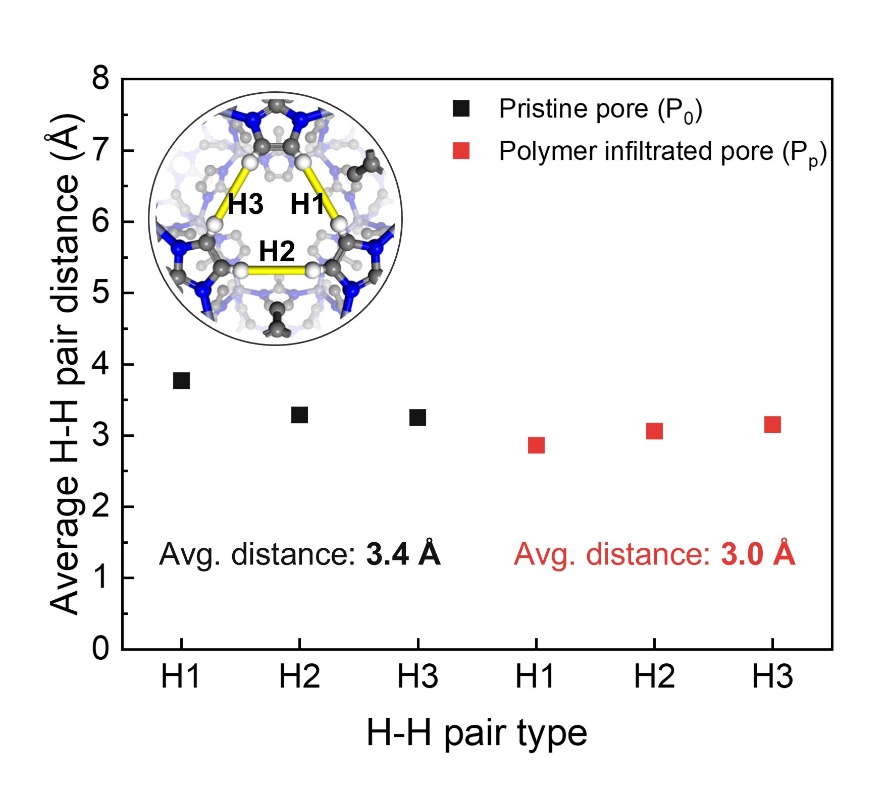


Figure S9. The average H–H pair distance in the polymer infiltrated pore


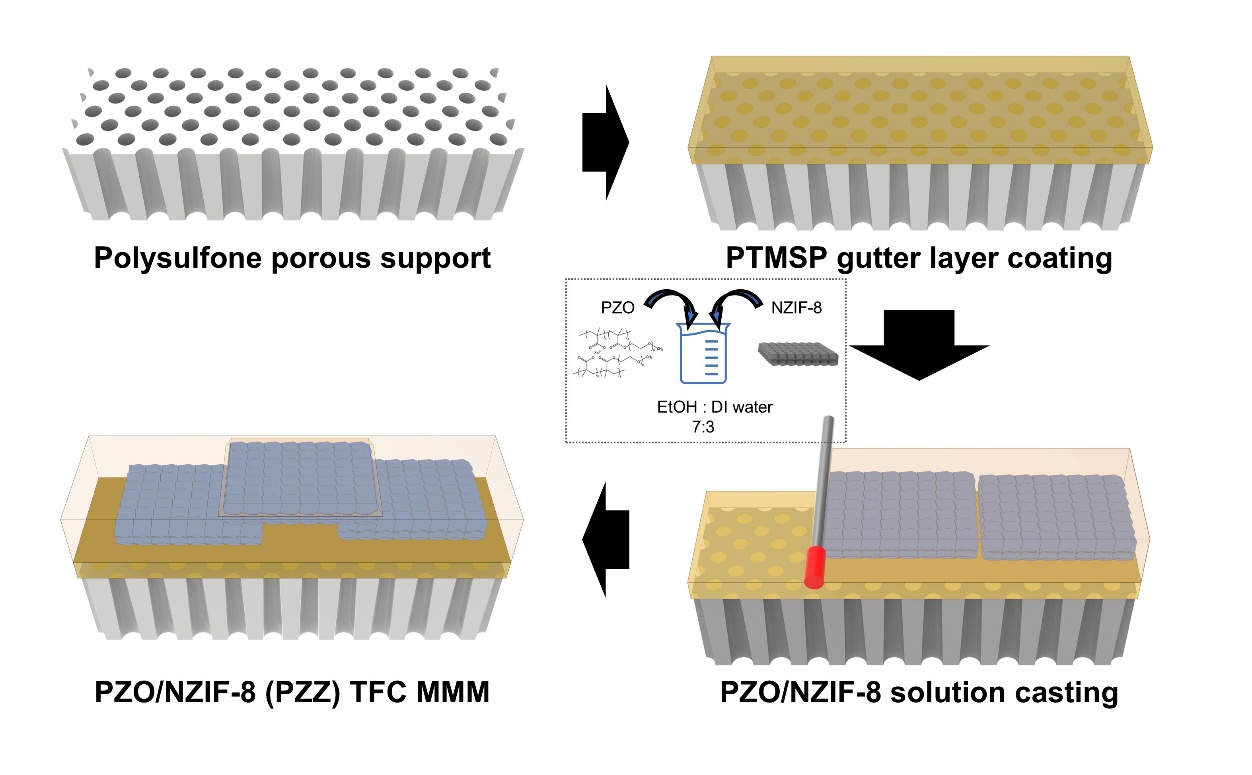


Figure S10. Fabrication procedure of PZZ TFC MMMs.


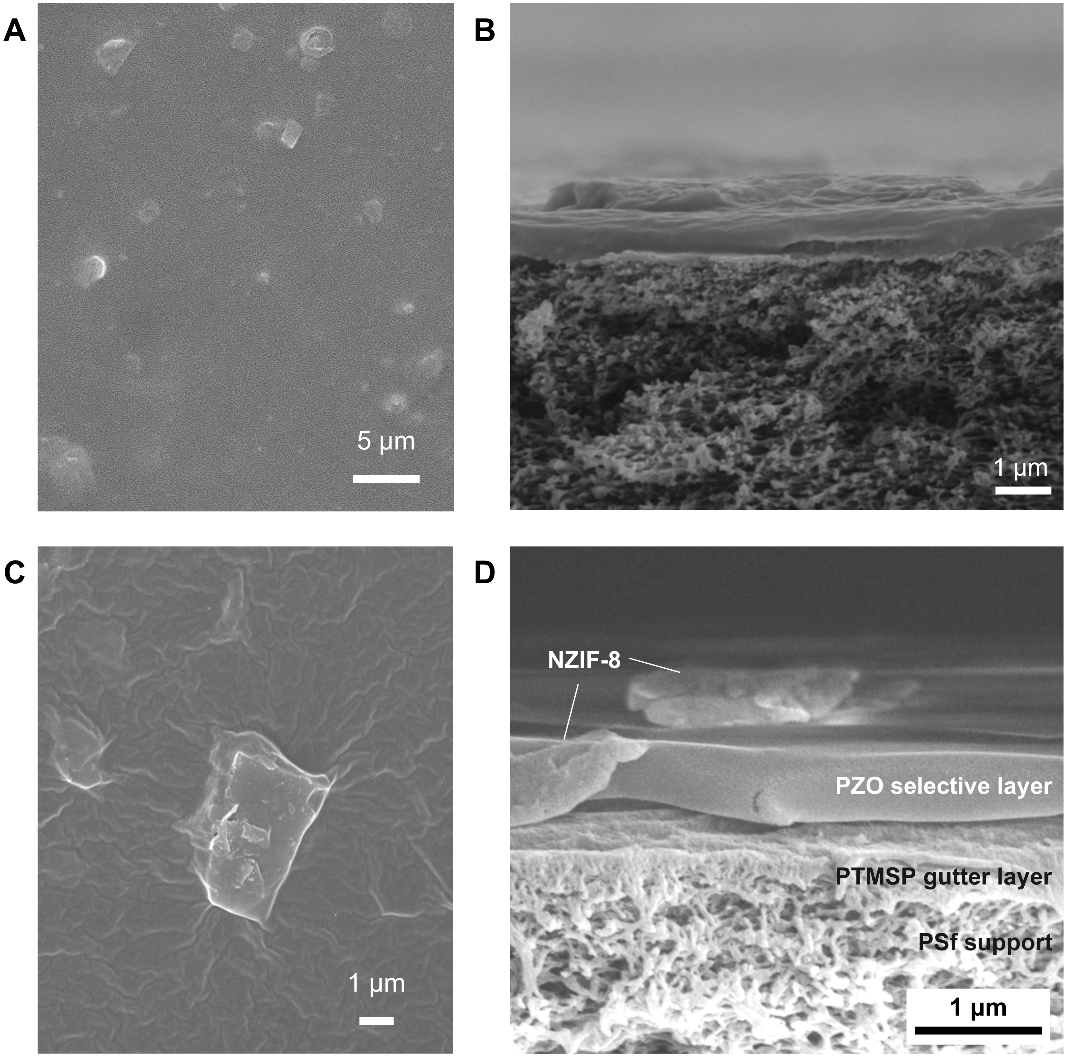


Figure S11. A-B, Low-magnification top-view and cross-section SEM images of the PZZ-10 TFC MMM. C-D, Corresponding high-magnification SEM images.


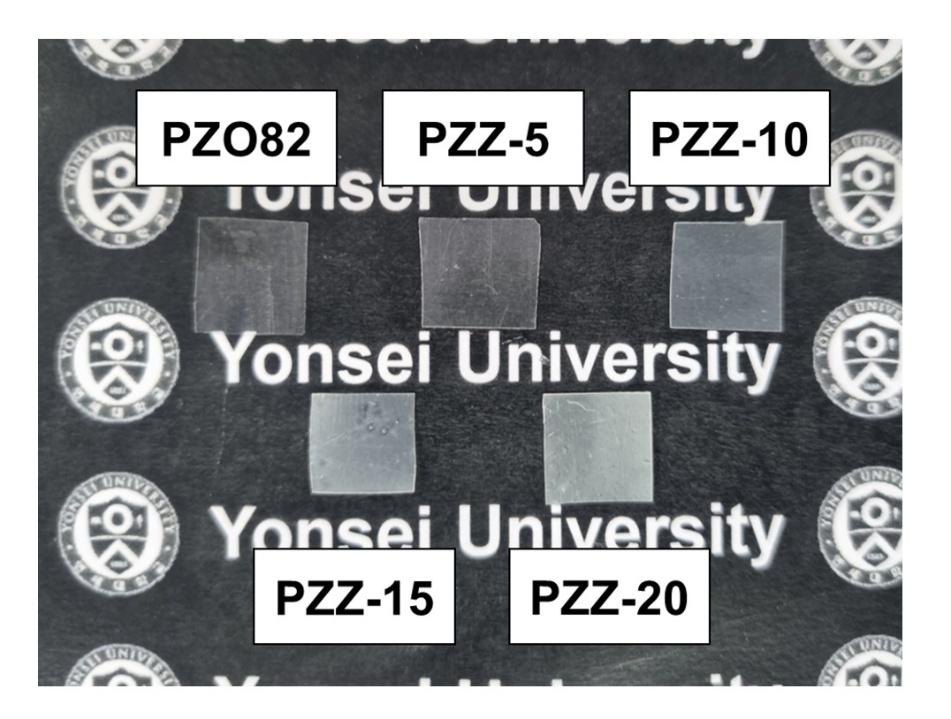


Figure S12. Photographs of the PZO copolymer and PZZ-x membranes.


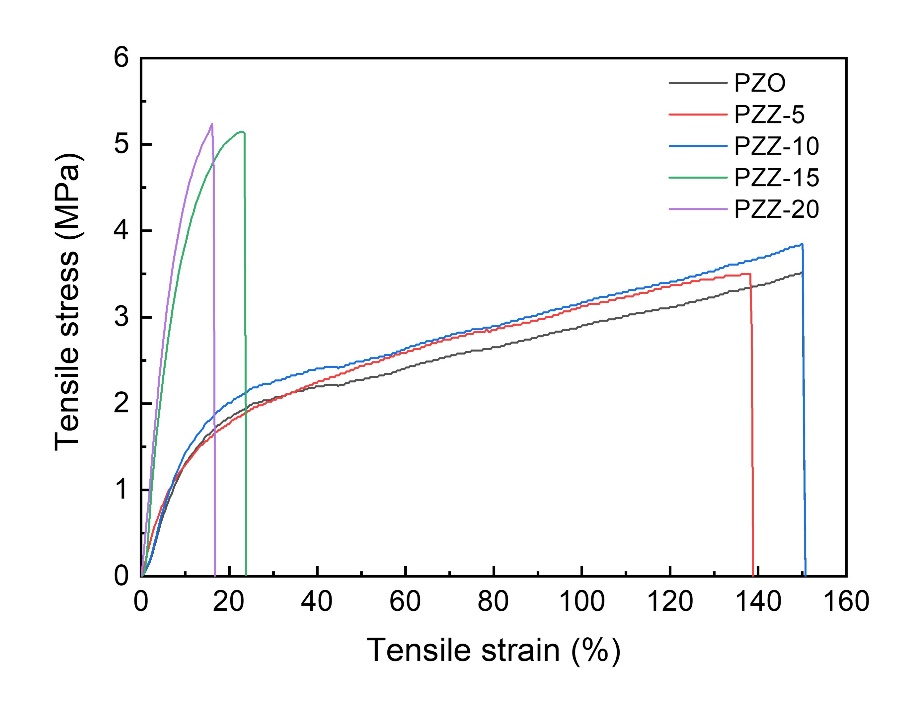


Figure S13. Stress-strain curves of PZZ-x membranes.


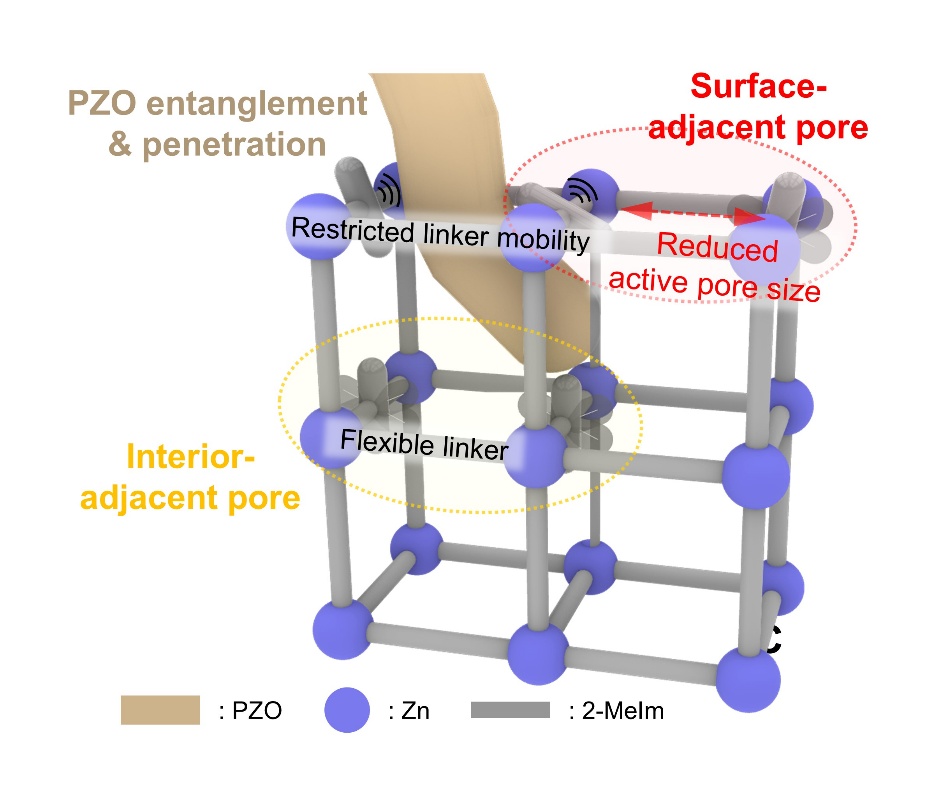


Figure S14. Schematic illustration of simplified PZO infiltrated ZIF-8 model, representing restricted linker mobility in surface-adjacent pore.


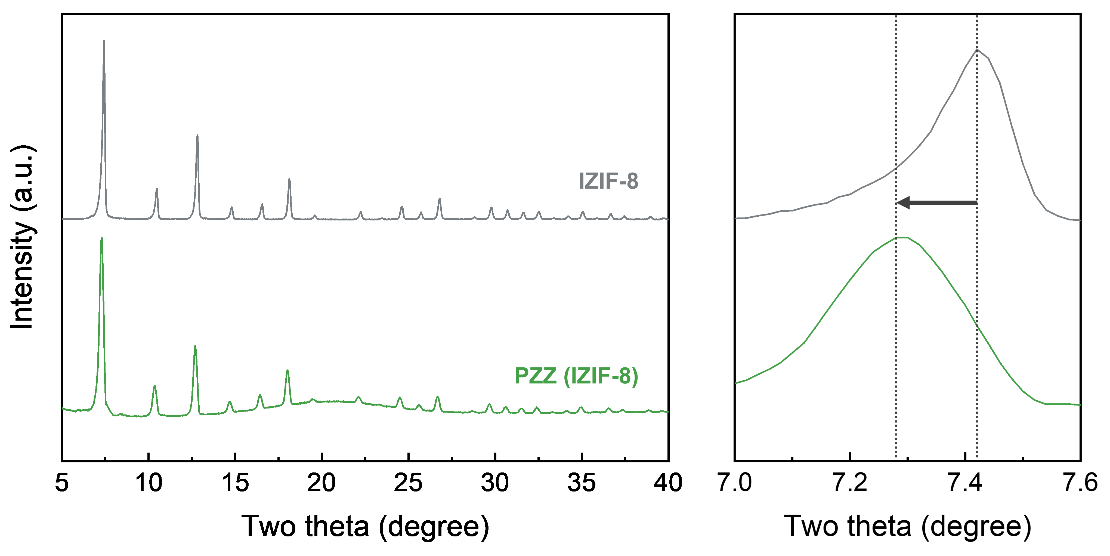


Figure S15. XRD pattern of IZIF-8-based MMM with magnified XRD patterns of the (011) peak.


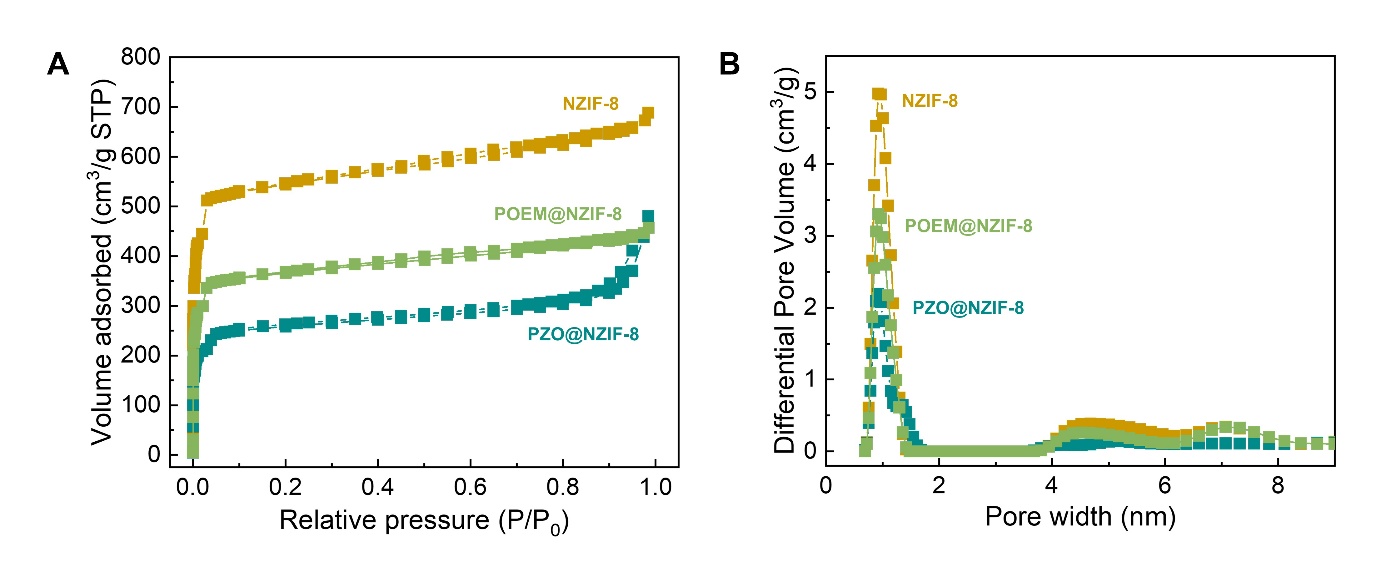


Figure S16. A, N_2_ adsorption-desorption isotherm and B, pore size distribution of NZIF-8, POEM@NZIF-8 and PZO@NZIF-8. measured at 77K. STP, Standard temperature and pressure.


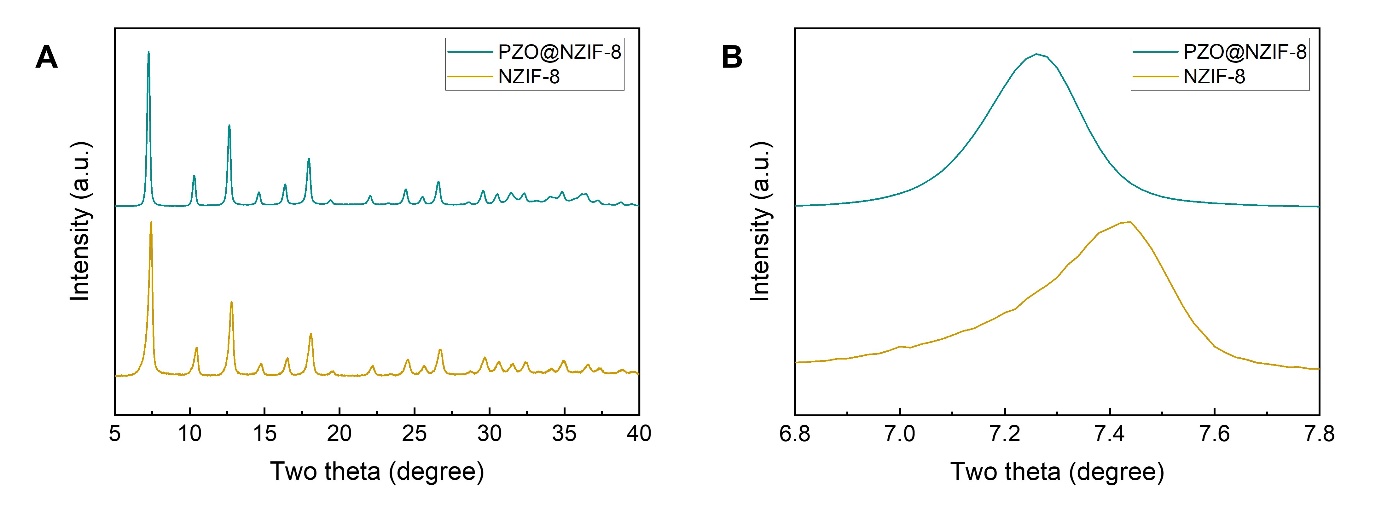


Figure S17. XRD patterns of PZO@NZIF-8 and NZIF-8. B, Magnified XRD patterns of (011) peak.


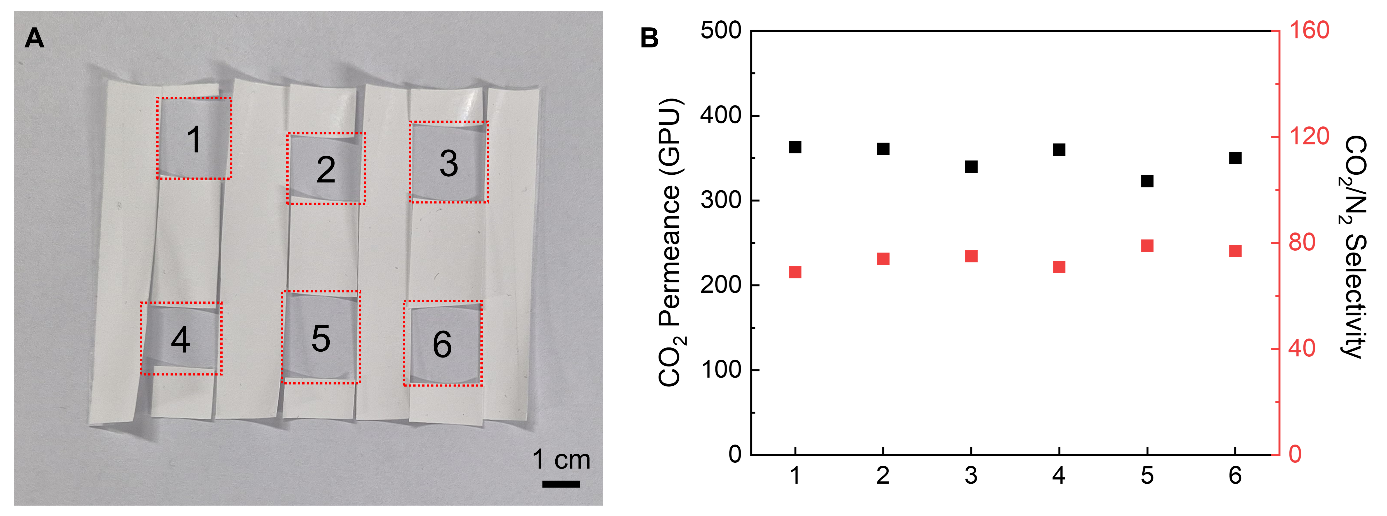


Figure S18. A, Photograph of the PZZ-10 TFC MMM showing multiple sampled regions. B, CO_2_ permeance and CO_2_/N_2_ selectivity of the corresponding sampled areas.


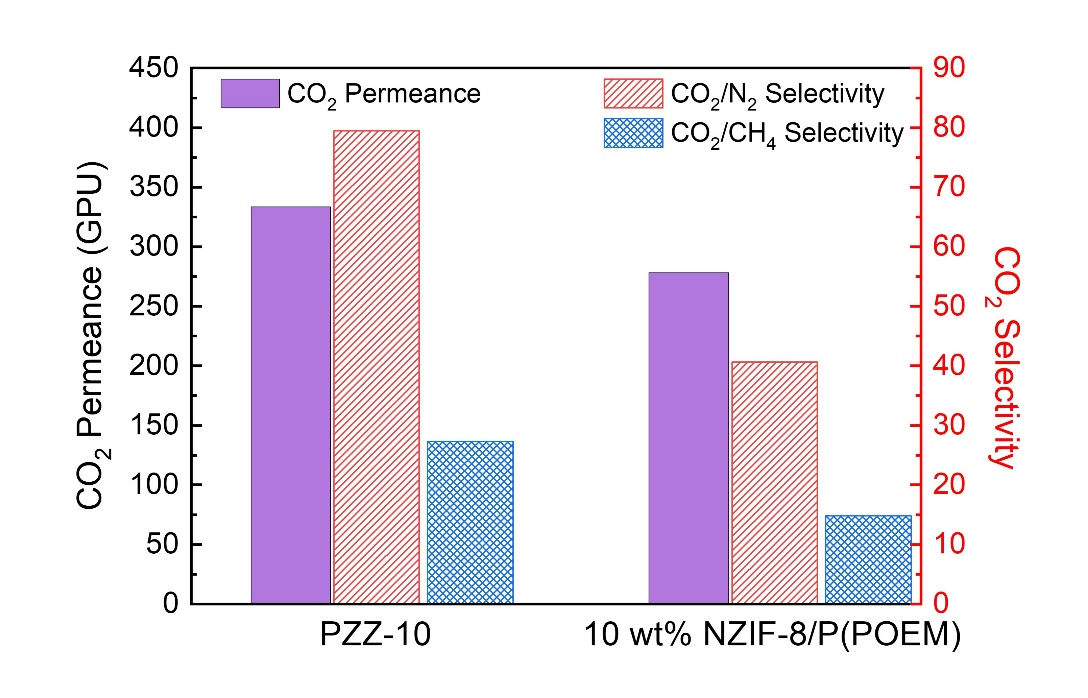


Figure S19. Single gas separation performances of PZZ-10 and 10 wt% NZIF-8/P(POEM) MMM.


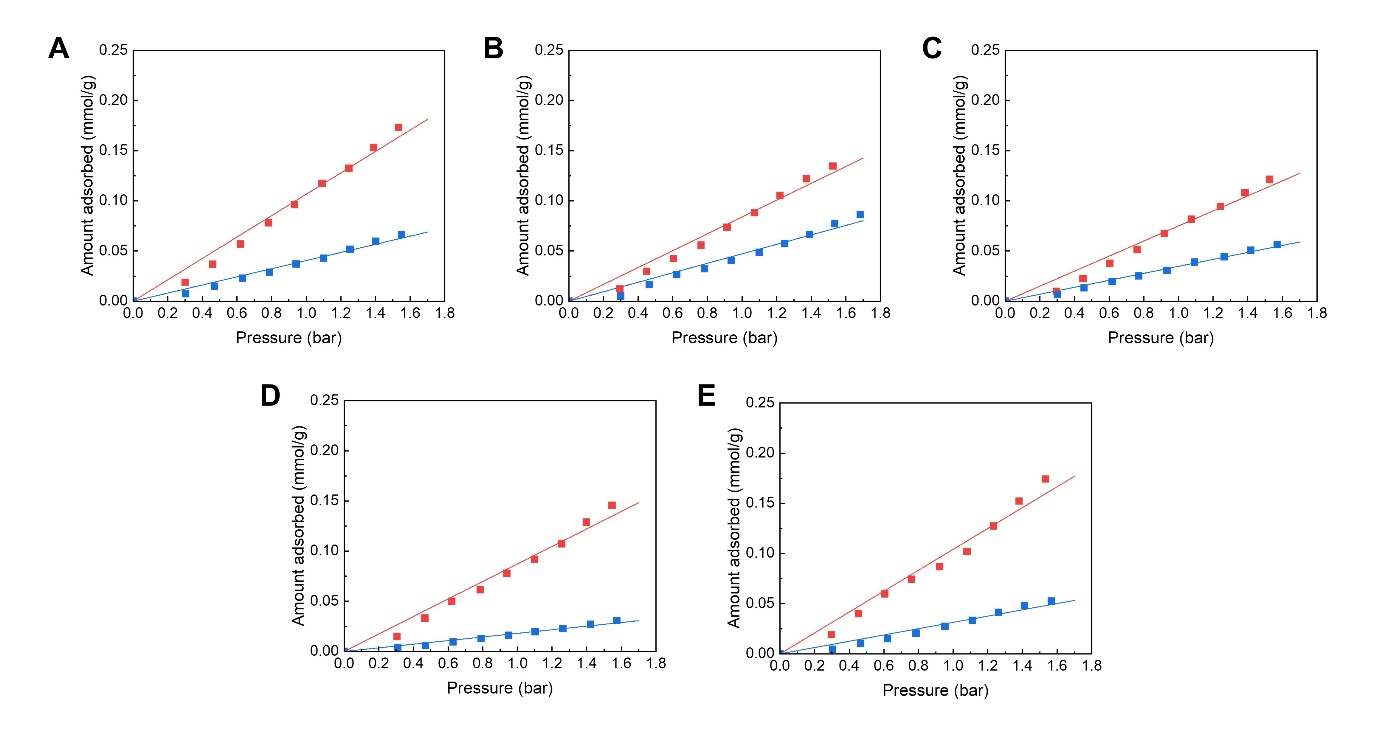


Figure S20. CO_2_ (red) and N_2_ (blue) adsorption isotherm curves of A, PZO, B, PZZ-5, C, PZZ-10, D, PZZ-15, and E, PZZ-20.


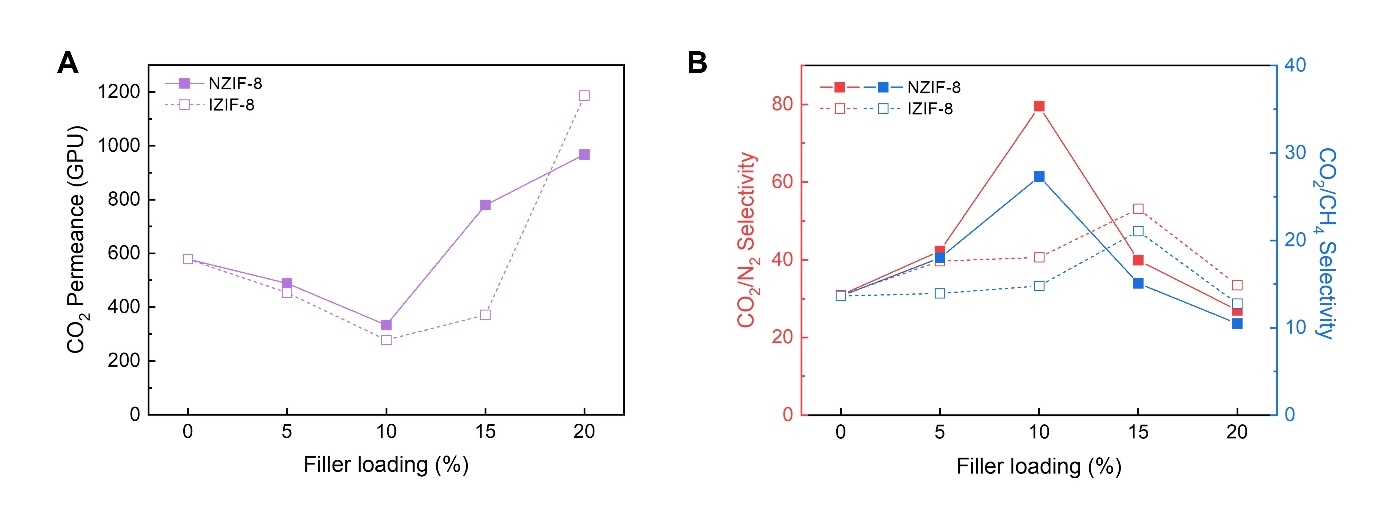


Figure S21. A, CO_2_ permeance and B, CO_2_/N_2_, CO_2_/CH_4_ selectivity of PZZ membranes with two different ZIF-8 morphology as a function of filler loading.


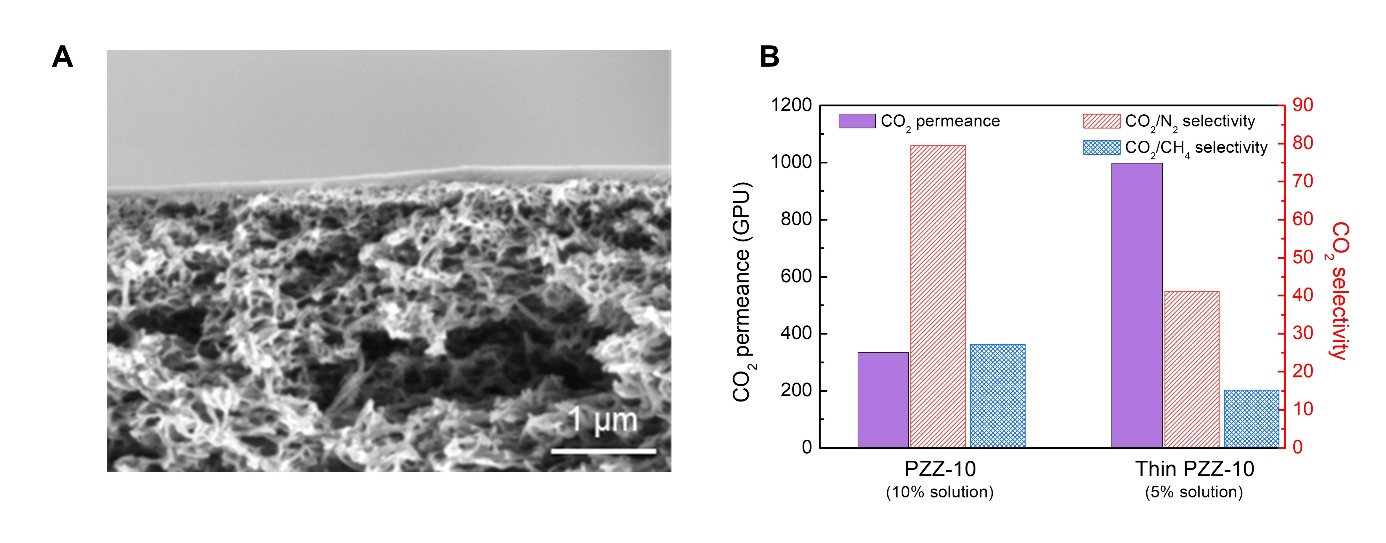


Figure S22. A, Cross-sectional SEM image of thin PZZ-10 membrane. B, Comparison of single gas separation performance of PZZ-10 and thin PZZ-10 membrane.


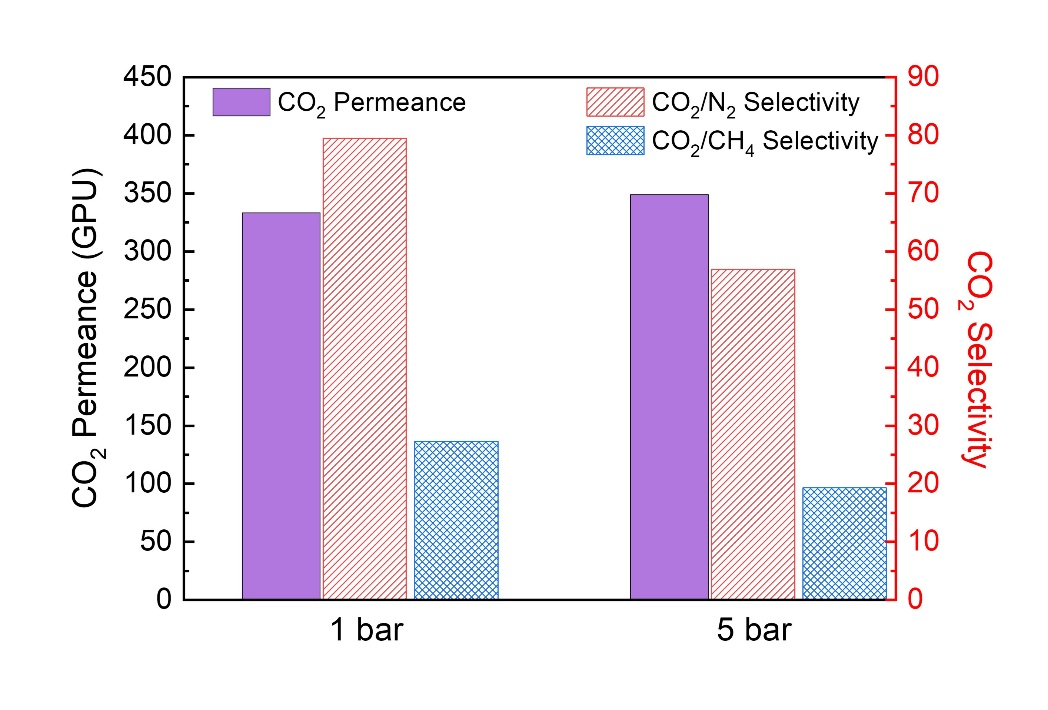


Figure S23. Single gas separation performance of PZZ-10 at 1 bar and 5 bar.


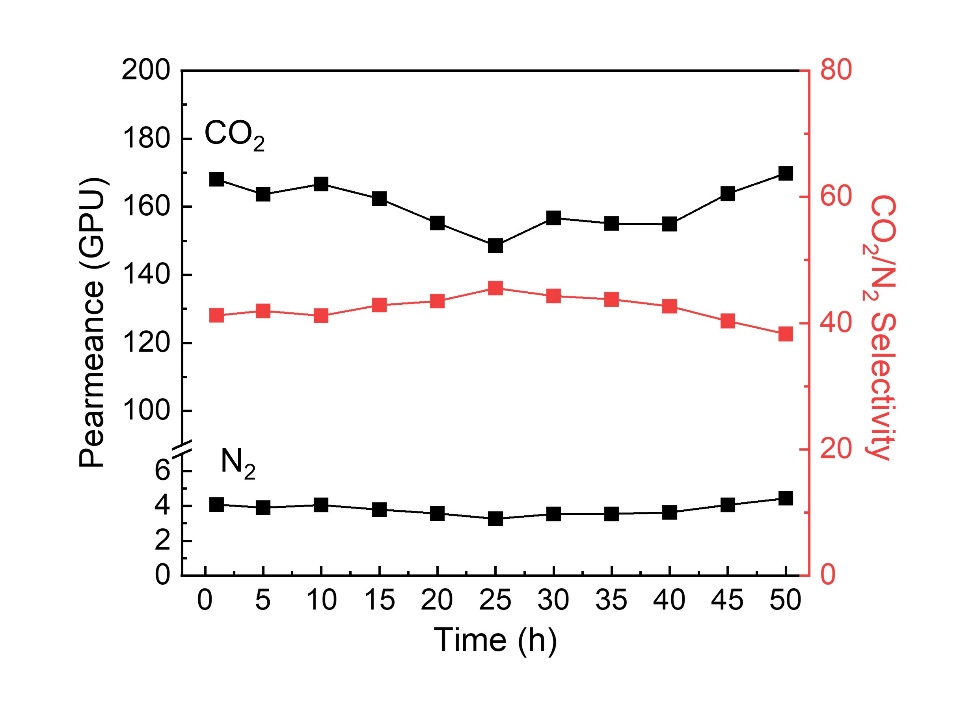


Figure S24. CO_2_/N_2_ separation test with humid conditions (Relative humidity = 85%) for 50 hours.

**
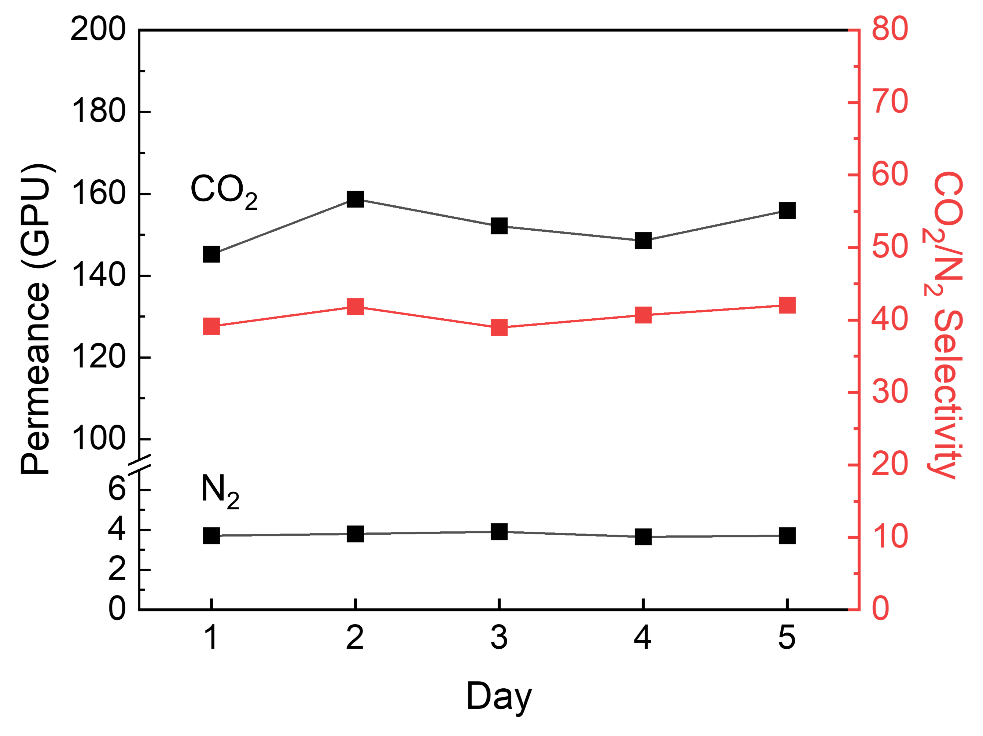
**

Figure S25. Long-term CO_2_/N_2_ separation test for 5 days. Tests were conducted at 1 bar, 25˚C with 15 vol% CO_2_: 85 vol% N_2_ mixture.

**
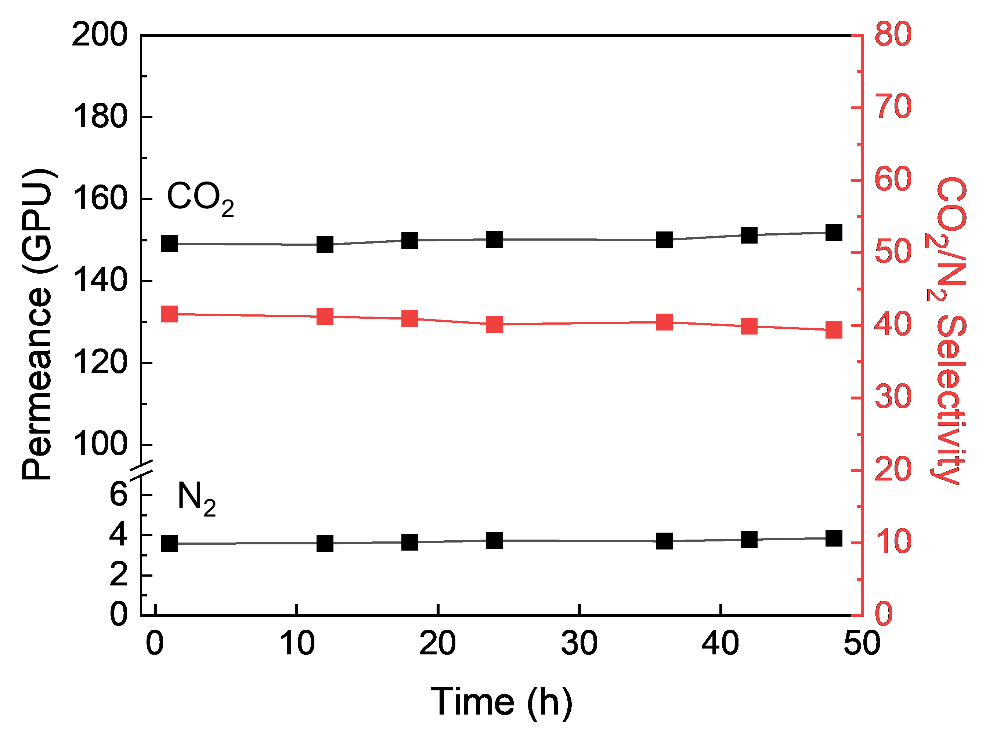
**

Figure S26. CO_2_/N_2_ separation test with 15 vol% CO_2_: 85 vol% N_2_ mixture and humid conditions (Relative humidity = 85%) for 48 hours.

**Table 1.** SEM-EDS data of weight percentage and atomic percentage of the elements in PZO copolymer.

| Element | wt% | wt% sigma | atomic % |
| --- | --- | --- | --- |
| C | 63.92 | 0.25 | 74.98 |
| O | 25.93 | 0.24 | 22.84 |
| Zn | 10.14 | 0.13 | 2.19 |
| Total | 100.00 |  | 100.00 |

**Table 2.** Gas separation performance of PZO and PZZ-x membranes.

| Sample | CO_2_ permeance (GPU) | N_2_ permeance (GPU) | CH_4_ permeance (GPU) | CO_2_/N_2_ Selectivity | CO_2_/CH_4_ Selectivity |
| --- | --- | --- | --- | --- | --- |
| PZO | 578.7 ± 2.1 | 19.2 ± 3.2 | 42.8 ± 4.1 | 30.9 ± 5.1 | 13.6 ± 1.3 |
| PZZ-5 | 488.4 ± 46.9 | 11.5 ± 0.4 | 27.3 ± 3.5 | 42.3 ± 2.4 | 18.0 ± 0.6 |
| PZZ-10 | 333.4 ± 18.2 | 4.2 ± 0.1 | 12.7 ± 2.8 | 79.5 ± 2.0 | 27.3 ± 4.6 |
| PZZ-15 | 779.3 ± 119.1 | 19.4 ± 1.3 | 51.5 ± 8.3 | 39.9 ± 3.4 | 15.1 ± 0.1 |
| PZZ-20 | 967.6 ± 7.9 | 35.9 ± 1.6 | 92.4 ± 3.1 | 27.0 ± 1.4 | 10.5 ± 0.4 |

**Table 3.** CO_2_/N_2_ separation performance of various membranes.

| **Membrane**  **Type** | **Material** | **CO_2_ permeance**  **(GPU)** | **CO_2_/N_2_**  **Selectivity** | **Ref.** |
| --- | --- | --- | --- | --- |
| **Polymer** | PEGBEM-g-POEM | 21.9 | 84.7 | ^[63]^ |
|  | PBEM-g-POEM | 29.3 | 73.3 | ^[64]^ |
|  | Pebax-1657 | 200 | 50 | ^[65]^ |
|  | HPEO | 850 | 37 | ^[66]^ |
| **MOF** | agZIF-UC-4 | 4752 | 36 | ^[59]^ |
|  | Atz-ZIF-8 | 690 | 29 | ^[60]^ |
|  | ZIF-L | 1200 | 14.5 | ^[61]^ |
|  | ZIF-8 | 1490 | 6.1 | ^[62]^ |
| **MMM** | ZIF-8/Pebax | 710 | 77 | ^[55]^ |
|  | MIP-202/Pebax | 936 | 52 | ^[56]^ |
|  | UTSA-16/PTO | 1070 | 41 | ^[23]^ |
|  | TAZIF-8/6FDA-DAM | 648 | 39 | ^[57]^ |
|  | MIL-140C/PGO | 1768 | 38 | ^[31]^ |
|  | ZIF-8/IL/PAP | 1017 | 33 | ^[32]^ |
|  | Ni, Co-MOF-74/PIM-1 | 1842 | 33 | ^[58]^ |
|  | PZZ-10 | 333 | 80 | This work |
